# Deploying digital health data to optimize influenza surveillance at national and local scales

**DOI:** 10.1371/journal.pcbi.1006020

**Published:** 2018-03-07

**Authors:** Elizabeth C. Lee, Ali Arab, Sandra M. Goldlust, Cécile Viboud, Bryan T. Grenfell, Shweta Bansal

**Affiliations:** 1 Department of Biology, Georgetown University, Washington, DC, United States of America; 2 Department of Mathematics & Statistics, Georgetown University, Washington, DC, United States of America; 3 Fogarty International Center, National Institutes of Health, Bethesda, Maryland, United States of America; 4 Department of Ecology & Evolutionary Biology and Woodrow Wilson School, Princeton University, Princeton, New Jersey, United States of America; The Pennsylvania State University, UNITED STATES

## Abstract

The surveillance of influenza activity is critical to early detection of epidemics and pandemics and the design of disease control strategies. Case reporting through a voluntary network of sentinel physicians is a commonly used method of passive surveillance for monitoring rates of influenza-like illness (ILI) worldwide. Despite its ubiquity, little attention has been given to the processes underlying the observation, collection, and spatial aggregation of sentinel surveillance data, and its subsequent effects on epidemiological understanding. We harnessed the high specificity of diagnosis codes in medical claims from a database that represented 2.5 billion visits from upwards of 120,000 United States healthcare providers each year. Among influenza seasons from 2002-2009 and the 2009 pandemic, we simulated limitations of sentinel surveillance systems such as low coverage and coarse spatial resolution, and performed Bayesian inference to probe the robustness of ecological inference and spatial prediction of disease burden. Our models suggest that a number of socio-environmental factors, in addition to local population interactions, state-specific health policies, as well as sampling effort may be responsible for the spatial patterns in U.S. sentinel ILI surveillance. In addition, we find that biases related to spatial aggregation were accentuated among areas with more heterogeneous disease risk, and sentinel systems designed with fixed reporting locations across seasons provided robust inference and prediction. With the growing availability of health-associated big data worldwide, our results suggest mechanisms for optimizing digital data streams to complement traditional surveillance in developed settings and enhance surveillance opportunities in developing countries.

## Author summary

Influenza contributes substantially to global morbidity and mortality each year, and epidemiological surveillance for influenza is typically conducted by sentinel physicians and health care providers recruited to report cases of influenza-like illness. While population coverage and representativeness, and geographic distribution are considered during sentinel provider recruitment, systems cannot always achieve these standards due to the administrative burdens of data collection. We present spatial estimates of influenza disease burden across United States counties by leveraging the volume and fine spatial resolution of medical claims data, and existing socio-environmental hypotheses about the determinants of influenza disease disease burden. Using medical claims as a testbed, this study adds to literature on the optimization of surveillance system design by considering conditions of limited reporting and spatial aggregation. We highlight the importance of considering sampling biases and reporting locations when interpreting surveillance data, and suggest that local mobility and regional policies may be critical to understanding the spatial distribution of reported influenza-like illness.

## Introduction

Seasonal influenza represents an important public health burden worldwide, and even within a single year, there is substantial variation in disease burden across populations [1–3]. On the other hand, pandemic influenza, which has the potential to cause millions of fatalities, is characterized by even more uncertainty in spatio-temporal risk. Traditional influenza surveillance is guided by the World Health Organization’s global standards for the collection of virological and epidemiological influenza surveillance data [4]. Epidemiological surveillance systems play an important role in our understanding of influenza dynamics and are used to identify seasonal influenza disease burden, severity, epidemic onset and seasonality, but they often suffer from reporting delays and limited, opportunistic sampling of the population.

Sentinel surveillance for influenza-like illness (ILI) is one such system that passively estimates influenza morbidity. Select general practitioners or health care facilities (“sentinels”) report aggregate counts of ILI to a centralized public health agency as an efficient means of collecting high quality data by focusing resources on a few population-representative sites [5]. The European Influenza Surveillance Network (EISN) collates sentinel ILI data from over 30 European countries, while the U.S. Centers for Disease Control and Prevention’s (CDC) ILINet surveillance system recruits roughly 2,000 sentinel physicians to submit reports on the percentage of patient visits with ILI weekly throughout the year [6–8]. While such sentinel surveillance systems are sufficient to provide situational awareness of national-level influenza activity, the coarseness of such data limit its use in local decision-making. Additionally, WHO recommends that choice in sentinel sites should consider population representativeness, geographical representation, patient volume, feasibility, and the data needs and goals of the surveillance system [4]. However, few ILI surveillance systems meet these criteria as they are limited by few incentives (e.g., data feedback from higher-level agencies, additional support for laboratory testing) and hampered by the administrative burden of data collection. Indeed, past studies have identified discrepancies across surveillance systems [9], and have investigated strategies to limit practitioner-based biases and improve capture of true population patterns in sentinel surveillance [10–12].

Medical claims represent an alternative potential source of passive ILI surveillance data with larger volume, fewer reporting delays, and finer spatio-temporal resolution than many traditional surveillance systems [13]. Additionally, medical claims data do not require additional administrative burden or voluntary reporting to a surveillance agency. We acknowledge that it may not be possible to combine these medical data streams directly into public health systems without further consideration of the ethical and privacy concerns of integrating health data at fine spatial resolutions [14]. In the meantime, however, we can leverage these features of medical claims and combine them with statistical models to explore the most informative design of passive surveillance systems and to test the robustness of ecological inference from opportunistic samples of health-associated big data.

We cannot, however, rely solely on the volume and resolution of big data to address surveillance data gaps; statistical models for ILI surveillance should also utilize information from known factors of spatial heterogeneity in influenza transmission and disease burden. Many studies have examined the relationship of environmental factors [15–21], transmission dynamics [22, 23], demography and contact patterns [24–32], immune landscapes [33, 34], and influenza type and subtype circulation [6, 29, 35–39] on influenza disease burden, although few have compared the relative importance of these mechanisms (except [40]). In addition, it is important to consider the possibility that individual patient behavior may bias the reporting of ILI disease burden, thus driving observed spatial heterogeneity. The association between poverty and social determinants [41–46], access to care, care-seeking behavior, and health insurance coverage [47–49], and reported ILI disease burden has been treated extensively elsewhere.

Surveillance system design may also contribute to the biased observation of ILI disease burden [11]. Current national sentinel systems in Australia, China, the United States, and Europe capture patients seen by 5% to less than 1% of active physicians in a given population, and while these systems strive to represent population demography, spatial distributions, and patient volume as accurately as possible, this is not always possible [4, 50, 51]. Theoretical work suggests influenza disease burden detection could be optimized if population coverage, care-seeking rates, and geographic access to care are considered in sentinel site choice [10, 11, 52, 53]. Non-traditional data with surveillance potential such as medical claims may enhance the estimation of attack rates through improved population coverage, better discriminate the duration of heightened epidemic activity and public health need through its real-time reporting, and improve our prediction of surge capacity needs with finer spatial resolution data. We note, however, that consideration of measurement biases is even more important as these digital data streams are opportunistic; they have greater volume and coverage in the population, but their measurement biases are less well studied [14]. Fortunately, non-traditional systems are often accompanied with metadata that provides context about the data coverage and user demographics, thus enabling explicit treatment of these potential flaws.

In this study, we developed a Bayesian hierarchical influenza surveillance model that accounts for transmission, environmental, influenza-specific, and socioeconomic factors, as well as measurement processes underlying spatial heterogeneity in reported influenza-like illness across counties in the United States. This model leveraged a large-scale and highly-resolved dataset of passive ILI surveillance from medical claims, and we validated the model results using ILI sentinel surveillance from CDC. Next, we probed the robustness of this ecological inference under limited data availability in order to mimic the potential conditions of real-world sentinel surveillance systems and to improve one primary goal of surveillance —the end-of-season estimation of disease burden. Our results highlight the relative contributions of surveillance data collection and socio-environmental processes to disease reporting, and emphasize the importance of considering surveillance system design and measurement biases when using surveillance data for epidemiological inference and prediction.

## Results

Using medical claims data representing 2.5 billion visits from upwards of 120,000 health care providers each year (see Methods: ‘Medical claims data’), we modeled influenza disease burden across U.S. counties for flu seasons from 2002-2003 through 2008-2009 and the 2009 pandemic using a hierarchical Bayesian modeling approach (see Methods: ‘Model structure’ and ‘Statistical analysis’). Our goal is to use this approach to simultaneously validate our surveillance data source and provide improved spatial surveillance of influenza burden based on socio-environmental and health behavior predictors. With these Bayesian models, we then study the impact of common surveillance limitations.

Our study considered six disease burden response variables: two measures of influenza disease burden (epidemic intensity and epidemic duration) in three populations (total population, children 5-19 years old, and adults 20-69 years old) across multiple seasons. We define *epidemic intensity* as a relative risk measure of population-normalized and detrended ILI activity above an epidemic baseline (details in Methods: ‘Defining influenza disease burden’). We define *epidemic duration* to be the number of weeks of ILI activity above an epidemic baseline. While total population models represent broad surveillance efforts to capture ILI activity in the community, the child and adult models may represent networks of school- or workplace-based surveillance systems. There were 13 county-level, 2 state-level and 4 HHS region-level predictors in the complete model (Table 1); all predictors were the same across response variables except care-seeking behavior, which was specific to the age group in the response (see Methods: ‘Predictor data collection and variable selection’). Analogous models considered influenza disease burden solely during the 2009 H1N1 pandemic. All model estimates of disease burden are openly available on GitHub at https://github.com/bansallab/optimize-flu-surveillance.

**Table 1:** Final model predictors, hypotheses, and data availability.

Factor	Index	Plot Label	Spatial Scale	Data Years	Hypothesized Effect
**Environmental factors**					
Influenza transmission	Specific humidity	humidity	county	2002-9	−
Respiratory disease risk	Fine particular matter	pollution	county	2003-9	+
**Transmission mechanisms**					
Density-dependent	Population density	popDensity	county	2002-9	+
Frequency-dependent	Average household size	householdSize	county	2002-9	+
**Diffusion mechanisms**					
Local spread	% child population	child	county	2002-9	+
Importation risk	% adult population	adult	county	2002-9	+
**Immunity**					
Vaccine-acquired	Toddler vacc. coverage	toddlerVacc	state	2003-9	−
	Elderly vacc. coverage	elderlyVacc	state	2002-7	−
Prior exposure	Population protected due to prior season exposure	priorImmunity	county	2003-9	−
**Influenza circulation**					
Dominant A subtype	% H3 subtype among flu type A samples	fluH3	HHS region	2002-9	+
B circulation	% B type among positive flu samples	fluB	HHS region	2002-9	+
H3 has older age distribution	adult population x dominant A subtype	adult-fluH3	HHS region	2002-9	+
B circulates primarily in children	child population x B circulation	child-fluB	HHS region	2002-9	+
**Socioeconomic factors and access to care**					
Health care availability	Hospitals per capita	hospAccess	county	2002-9	+
Social deprivation	% single-person households	onePersonHH	county	2005-9	+
Material deprivation	% in poverty	poverty	county	2002-9	+
Claims-reporting population	% with health insurance	insured	county	2002-9	+
**Measurement factors**					
Claims database coverage	% physicians reporting to claims database	claimsCoverage	county	2002-9	+
Care-seeking behavior in claims database	All visits per capita reported in database	careseeking	county	2002-9	+

### Surveillance model validation

We validated our surveillance models of medical claims data in two ways. First we compared the model fits to CDC ILI and laboratory-confirmed surveillance data (details in Methods: ‘Model assessment and validation’). We then verified that significant socio-environmental factors identified by our models are consistent with past influenza studies.

#### Comparison to CDC surveillance data

We compared epidemic intensity surveillance model fits to CDC surveillance for ILI (*ILINet*) and laboratory-confirmed influenza (*NREVSS*) across HHS regions for all seven seasonal influenza seasons in our study period. There was a moderate linear correlation between total population model fits and positive laboratory confirmation numbers from *NREVSS*. Additionally, we found a moderate linear correlation between total, children, and adult model fits and the percentage of patients (in the appropriate age group) observed with ILI from *ILINet*. Supplementary details on the results and methodology may be found in Section 2.4 in S1 Appendix.

#### Socio-environmental determinants of disease burden

Here, we report the association between two measures of disease burden, epidemic intensity and epidemic duration, and the transmission, environmental, influenza-specific, and socioeconomic determinants of disease risk. Our work is one of the first large-scale studies to examine wide-ranging hypotheses on the effect of these factors on influenza rates, and our results are consistent with previous evidence related to specific humidity, household contact, and age and subtype interactions, which serve to validate our model [6, 15, 17, 18, 20, 29, 35–40]. Additional models linking influenza A/H3 and B circulation with adult and child epidemic intensity, respectively, also align with our understanding of the age distribution of disease risk [6, 29, 35–39]. Full results on socio-environmental determinants for intensity among children and adults and during the 2009 H1N1 pandemic can be found in Section 5 and Section 2.5 in S1 Appendix, respectively.

**Fig 1:**
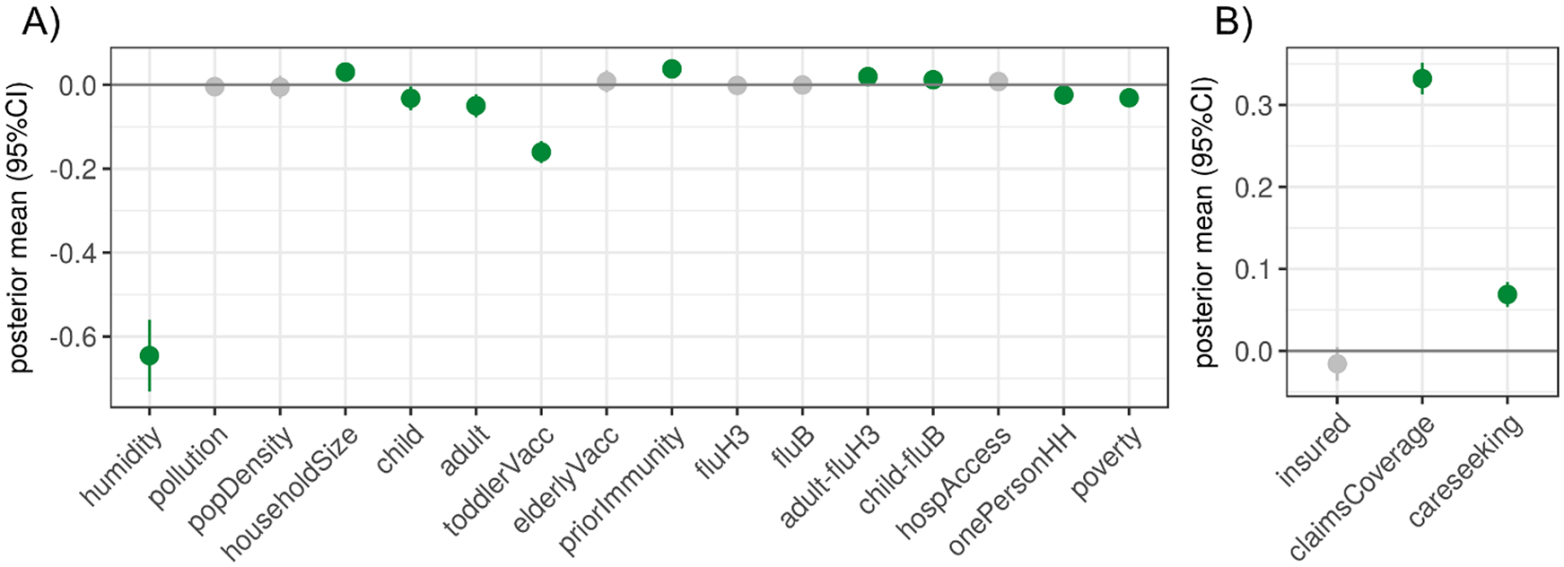
Socio-environmental and measurement factors associated with the epidemic intensity surveillance model. For the total population multi-season epidemic intensity models, these are the means and 95% credible intervals for the posterior distributions of the A) socio-environmental coefficients and B) measurement-related coefficients. Distributions indicated in green were statistically significant (95% credible interval deviated from zero). Coefficients are reported according to their effect on log epidemic intensity.

Epidemic intensity had positive associations with average household size, prior immunity (a proxy calculated from intensity in the previous influenza season, influenza type and subtype distribution, and membership in the same antigenic cluster or lineage), and the adult:%H3 and child:%B interaction terms (Fig 1A). There were negative associations with adult and child population sizes, specific humidity, poverty, single person households, and toddler vaccination coverage. A coefficient mean of -0.65 for humidity means that for every unit increase in specific humidity, on average, there is a 47.8% decrease in epidemic intensity (a -0.65 change in log epidemic intensity) if all other predictors remain constant (N.B., A unit is a standard deviation in the original scale of the predictor, given that predictors are centered and standardized).

Epidemic duration had positive associations with the interaction between influenza B circulation and child population size, influenza B circulation, estimated average household size, population density, a proxy for prior immunity, and elderly vaccination coverage (Fig AB in S1 Appendix). There were negative associations with H3 circulation among influenza A, average flu season specific humidity, toddler vaccination coverage, and proportion of the population in poverty.

#### Measurement factors affect disease burden

Our model incorporated factors that may alter the observation of ILI disease burden in our medical claims dataset, thus enabling us to identify the size and directionality of these biases. We found that care-seeking behavior and claims database coverage had strong positive associations with epidemic intensity (Fig 1B). Care-seeking behavior and claims database coverage also had strong positive associations with epidemic duration (Fig AC in S1 Appendix). A coefficient mean of 0.13 for database coverage means that for every standard deviation increase in database coverage, on average, there is a *e*^0.13^ ≈1.14 week increase in epidemic duration if all other predictors remain constant.

### Influenza surveillance and spatiotemporal patterns

The outputs of our statistical models provide improved surveillance of U.S. county-level disease burden due to influenza-like illness from 2002 to 2010. In this section, we explore broad temporal and spatial trends of seasonal ILI, the burden of seasonal ILI among children and adults, and the burden of ILI during the 2009 H1N1 pandemic.

#### Epidemic intensity

We found that county spatial dependence (i.e., neighborhood structure) and state group effects captured most of the variability in the total population epidemic intensity observations, when comparing the estimated precision terms across the various group effects in our model. The variability explained by these two terms was followed by the terms for region, season, observation error, and county, respectively (Table A in S1 Appendix).

**Fig 2:**
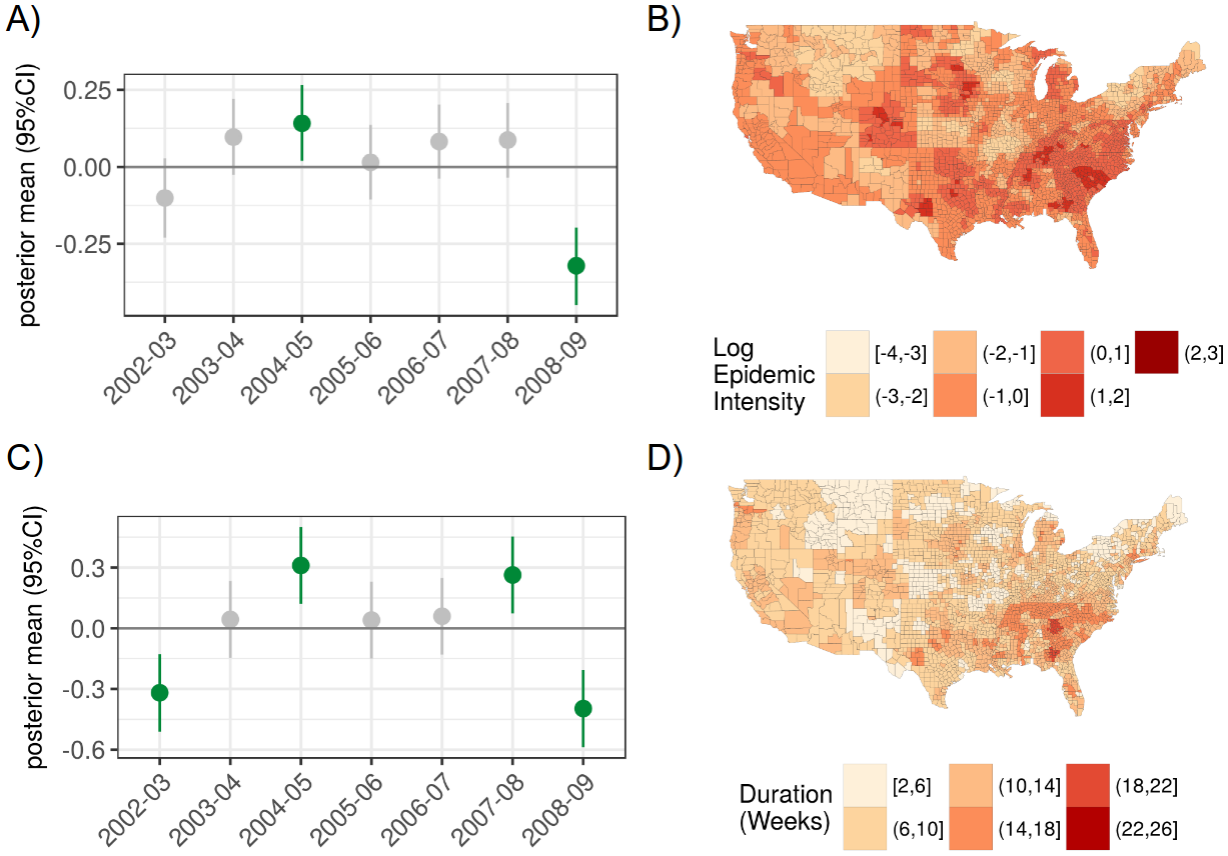
Temporal and spatial group effects for the epidemic intensity and epidemic duration surveillance models. A) The posterior mean and 95% credible intervals for group (random) effects are shown for log epidemic intensity. B) Continental U.S. county map for fitted log epidemic intensity for an example flu season (2006-2007). C) The posterior mean and 95% credible intervals for group (random) effects are shown for log epidemic duration. D) Continental U.S. county map for fitted epidemic duration for an example flu season (2006-2007).

Group (random) effects were used to identify consistent spatial or temporal patterns across locations and study years. We found that the 2004-2005 flu season had greater intensity (estimate of 0.14 translates into *e*^0.14^ ≈1.2 times greater risk of disease than other seasons), while 2008-2009 had relatively low intensity (estimate of -0.32 translates into *e*^−0.32^ ≈0.73 times lower risk of disease than other seasons) (Fig 2). For the epidemic intensity model, no single region had a significant group effect, although several South Atlantic states like Georgia, Maryland, North Carolina, South Carolina, Tennessee, and Virginia had relatively greater risk than other states across the study period (on average, ≈1.79 greater risk than other states), while several Plains and Rocky Mountain states like Kansas, Minnesota, Missouri, Montana, and Utah had relatively lower risk (on average, ≈0.62 lower risk than other states) (Fig 2, Fig C in S1 Appendix). A log epidemic intensity of 0 indicates that the location matched the expectation for a given flu season; a log epidemic intensity of 1 indicates that the location had *e*^1^ ≈2.72 times more disease than the expectation.

#### Epidemic duration

Among the components of the epidemic duration model, county spatial dependence, observation error, and season, followed by those among state, region, and county, explained the most variability in the data respectively (Table D in S1 Appendix).

We found that the 2004-2005 and 2007-2008 seasons had relatively long epidemic periods while the 2002-2003 and 2008-2009 seasons had relatively short epidemics (Fig 2). At the region-level, the Atlanta region (HHS Region 4) had longer epidemics (*e*^.2^ ≈ 1.22 times the length of those in other regions) (Fig AE and Fig AF in S1 Appendix).

#### Epidemic intensity among children and adults

We examined the spatial trends for models of ILI epidemic intensity among children and adults as representations of school-based and workplace-based models of ILI surveillance (Fig AJ and Fig AK in S1 Appendix). Similar to the total population epidemic intensity models, several South Atlantic states like Virginia, Tennessee, North Carolina, South Carolina, and Georgia had relatively greater risk across both children and adults than other states in the study period (Fig AL and Fig AM in S1 Appendix).

#### Epidemic intensity during the 2009 H1N1 pandemic

We examined the spatial trends for ILI epidemic intensity during the fall wave (August 2009 to January 2010) of the 2009 H1N1 pandemic (Section 2.5 and Fig N in S1 Appendix). State group effects explain much of the variation in this model, and mid-Atlantic states like Maryland, West Virginia, Virginia, Kentucky, Tennessee, North Carolina, and South Carolina had relatively high intensity during the pandemic (*e*^0.62^ ≈ 1.89 times greater risk than other states) (Fig M in S1 Appendix).

### Sentinel surveillance design

Leveraging the large volume and spatial resolution of our data, we sought to examine the robustness of our model predictions and inference in order to assess their suitability for disease surveillance and prediction. First, we compared our estimation of epidemic intensity when using analogous models at the county and state spatial units of analysis. These comparisons recall hypothetical scenarios where inference from state-level surveillance data might inform county-level decision making in the absence of resolved county-level data. Next, two model sequences were designed to simulate different flu sentinel surveillance systems —*fixed-location sentinels*, where the same sentinel locations reported data every year, and *moving-location sentinels*, where new sentinel locations are recruited each year. A third model sequence considered the specificity of inference and model predictions to certain *inclusion of historical data*, thus providing insight into the generalization of our model to epidemic forecasting. We examine these applications for the total population epidemic intensity model, and ten replicates were performed for each model with missingness to generalize findings beyond that of random chance.

#### Comparison of county and state spatial units of analysis

**Fig 3:**
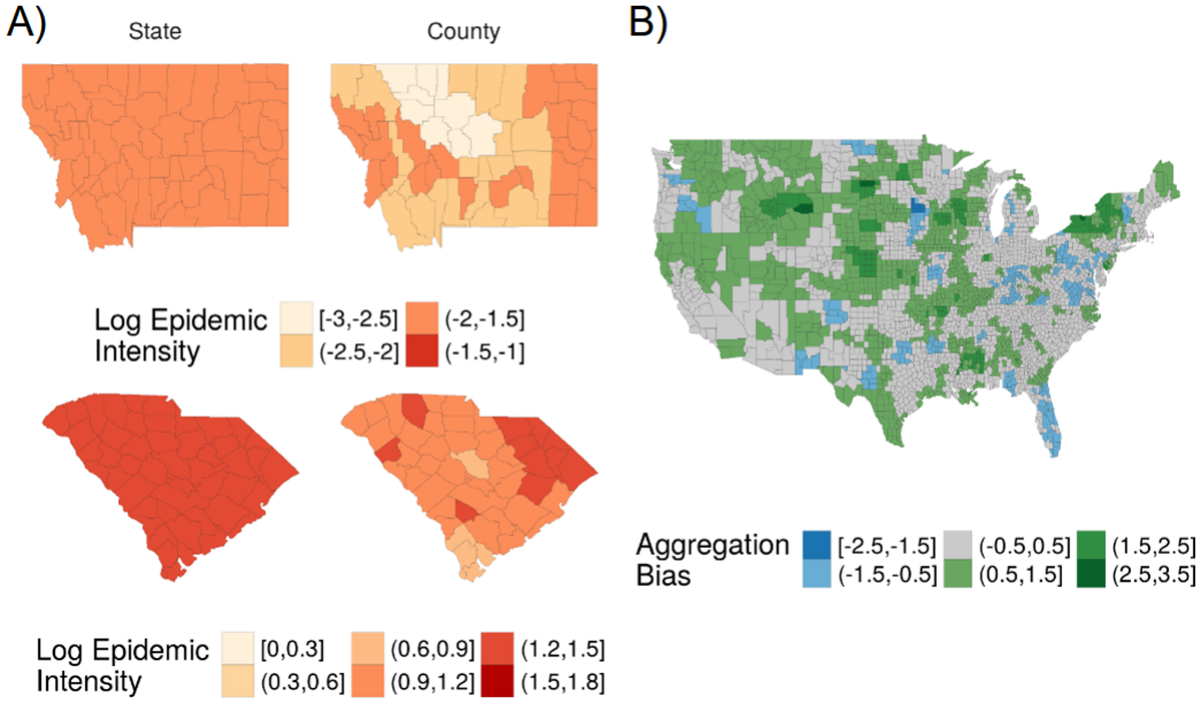
Discrepancies between state and county surveillance models for epidemic intensity. A) Comparison of state and county surveillance models (left and right columns, respectively) for log epidemic intensity for states with overestimation and underestimation with the state surveillance model —Montana (top row) and South Carolina (bottom row), respectively. B) Aggregation bias between county and state epidemic intensity surveillance models for the 2006-2007 influenza season, where error is defined as the difference between fitted values for county and state log epidemic intensity. Negative error (blue) indicates that the state-level surveillance model underestimated risk relative to the county-level surveillance model, and vice versa.

We compared analogous state-level and county-level epidemic intensity model outputs to examine the added value of county-level information on ILI surveillance (Fig 3, Fig P in S1 Appendix). Negative error indicates that the state surveillance model underestimates risk relative the county surveillance model, and vice versa. An error of -1 means that state surveillance model risk was *e*^−1^ ≈0.37 of the county model risk, while an error of 1 means that the state risk was *e*^1^ ≈2.72 of the county model risk. The state-level surveillance model captured high risk areas like the South Atlantic quite well, with some underestimation of ILI risk relative to the county-level surveillance model. Plains and Rocky Mountain states, typically low risk areas, were overestimated substantially in the state surveillance model relative to that of the county. States with a larger absolute discrepancy between county and state surveillance model fits (labeled here as “aggregation bias”) have greater within-state heterogeneity in epidemic intensity (Fig Q in S1 Appendix).

#### Sentinels in fixed locations

**Fig 4:**
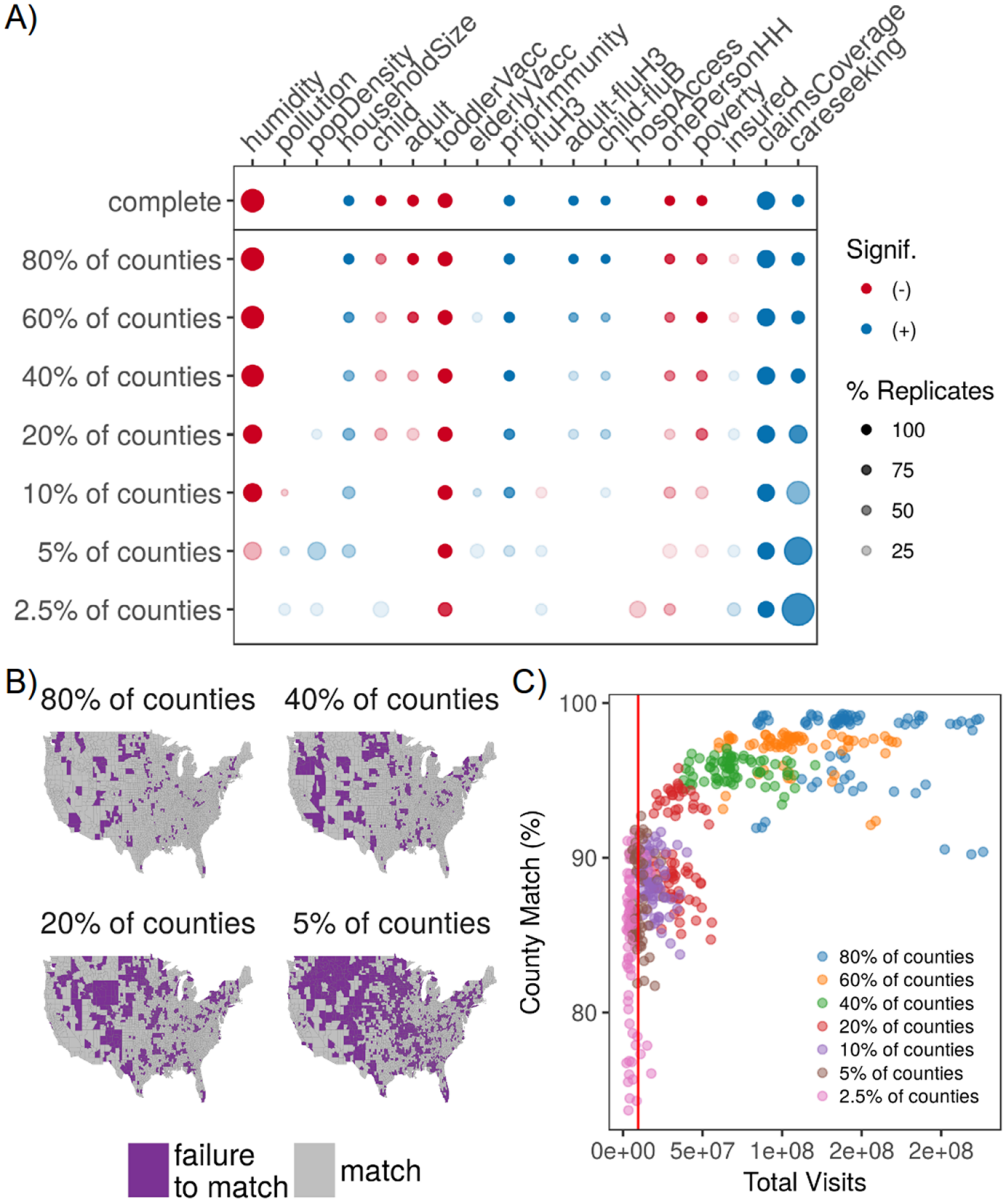
Optimizing design of sentinel surveillance systems. A) Diagram indicating changes to model inference as fewer fixed-location sentinels reported data. Color indicates directionality of the significant effect (blue is positive, red is negative) while greater transparency indicates a lower percentage of replicates with a significant effect (for models with missingness); dot size represents the magnitude of the posterior mean (or average of the posterior mean across replicates). Predictors with no significant effect across the sequence of models were removed for viewing ease, and absence of a dot means the effect was not significant across any replicates. B) Map of model prediction match between the complete model and the 80, 40, 20, and 5% reporting levels for fixed-location sentinels. Match between the complete and sentinel models were aggregated across 70 season-replicate combinations (7 seasons * 10 replicates). Color indicates match between posterior predictions in the missing and complete models (purple represents a failure to match in at least 10% of season-replicate combinations). Failure to match means that the interquartile ranges for two posterior distributions failed to overlap with each other C) Scatterplot of county match percentage between the complete and sentinel models versus the total volume of medical claims visits. Each point represents a single season-replicate combination, and colors represent the reporting level of the fixed-location sentinels. The dashed line indicates the average visit volume in CDC’s ILINet during the study period, and it corresponds roughly with the 5% reporting level for our medical claims database.

In this sequence of seven models, we removed 20, 40, 60, 80, 90, 95, and 97.5% of randomly selected county observations across all years. There was a strong linear relationship between true observed and fitted values when using these model replicates for out-of-sample validation (Pearson’s R = 0.56), and observations with poor fits seemed isolated to a few counties across all levels of missingness (Fig W in S1 Appendix). We also found that the effect sizes of determinants were pulled towards zero as fewer sentinel counties reported ILI epidemic intensity, but the primary conclusions remained robust. We noted that the positive effect of care-seeking increased across most model replicates as fewer sentinels reported data (Fig 4A). Model predictions (county-season fitted values) remained quite robust relative to the complete model, even when 80% of counties were excluded (Fig 4B). Across different levels of missingness and seasons, we examined the association between visit volume contributing to model fit and county match percentage (Fig 4C). Match percentage increased and match percentage variance declined as the volume of contributing visits increased. The volume of visits captured by CDC’s ILINet represents roughly 5% of the medical claims database and remains in the high variance range for county match.

#### Sentinels in moving locations

In this sequence of four models, 20, 40, 60, and 80% of randomly chosen seasonally-stratified observations were removed. Similar to the fixed-location sequence, there was a strong linear relationship between true observed and fitted values when using these model replicates for out-of-sample validation (Pearson’s R = 0.81), and observations with poor fits seemed isolated to a few counties (Fig X in S1 Appendix). Predictor effects were pulled towards zero as fewer sentinel counties reported ILI, the effects with the smallest means were pulled towards zero and predictors with no effect in the complete model were found to be significant (Fig S and Fig T in S1 Appendix). Model predictions had good agreement with the complete model up to a threshold between 60 and 80% missingness, where the model no longer provided reasonable fits.

#### Inclusion of historical data

In this sequence of models, one, three, and five out of seven flu seasons in the study period were completely removed. While there was a moderately strong linear relationship in out-of-sample validation (Pearson’s R = 0.62), many observations in the 2005-2006 seemed to be overestimated in the model (Fig Y in S1 Appendix). The effect of determinants changed substantially when more than one season was removed, particularly when they had small effect sizes in the complete model (Fig U and Fig V in S1 Appendix). Notably, medical claims coverage and care-seeking were two of three predictors that remained consistent in the magnitude and direction of inference across all model replicates. Model predictions were robust relative to the complete model only when one season was removed. Beyond that, many seasonal fitted values were poor, particularly for some seasons where data had been removed.

## Discussion

Reliable surveillance systems are at the heart of public health preparedness, mitigation and response. In this study, we opportunistically use an administrative data source to inform influenza spatio-temporal patterns and surveillance design. Our medical claims data represented an average of 24% of all U.S. health care visits to approximately 37% of all health care providers across 95% of U.S. counties during flu season months in our study period (increasing to 38%, 70% and 96%, respectively, by 2009). We pair these data with a Bayesian hierarchical modeling approach which enables “borrowing information”, the efficient incorporation of spatial dependence and group indicators for spatial and temporal random effects. The high resolution and coverage of our data combined with this spatial statistical approach allowed us to contribute to influenza surveillance in three ways: (a) enhance fine-grain mapping of disease burden from influenza-like illness to guide local influenza preparedness and control; (b) inform the future treatment of digital data streams as a measurement process for infectious disease surveillance; and (c) systematically explore surveillance design choices. Moreover, our surveillance model enables the generation of synthetic datasets that capture realistic spatial distributions of ILI, which can be used in models to inform the design of control strategies and surveillance systems.

In the process of our model validation, we also consider the relative importance of 16 environmental, demographic, or socio-economic factors in predicting influenza spatial heterogeneity. This makes ours the first large-scale influenza study to simultaneously consider multiple hypotheses across spatial scales (with the exception of work in review by Chattopadhyay et al [40]), and generates a new set of hypotheses on drivers of influenza spread. Our results strengthen the epidemiological link between humidity and influenza transmission and survival in temperate regions by finding strong negative associations between absolute humidity and both epidemic intensity and duration [54, 55]. These associations were not simply influenced by the strong spatial dependence of humidity —the relative effect of this predictor remained consistent when we removed the model’s spatial dependence term (Section 2.3 in S1 Appendix) and considered humidity as the sole model predictor (Fig AO in S1 Appendix). Charu et al. suggests that humidity may not provide additional information beyond a well-calibrated model of human mobility [56], and our work suggests that humidity among other factors is necessary to capture the end-of-season spatial heterogeneity in influenza disease burden. We also observed that higher estimated prior immunity was associated with greater epidemic intensity and longer epidemic durations. As larger epidemics induce more antigenic drift in subsequent seasons, we suggest that this drift renews population susceptibility every season, even on small spatial scales [57]. Finally, while higher vaccination coverage among toddlers was associated with lower epidemic intensity, we were surprised to note that higher vaccination coverage among elderly was associated with longer epidemics.

While statistical results may be neither interpreted as causative evidence nor are free from the possibility of spurious associations, future validations of our findings on influenza epidemiology will become more possible as high volume data sources achieve wider availability and tests of multiple hypotheses become more prevalent [40]. From the perspective of surveillance operations, we acknowledge the limitations of including many predictors with disparate data sources in our model; nevertheless, we gained additional epidemiological knowledge from the multiple predictor comparisons and note that all of the data we used were publicly available annually and at the county scale. In the future, comparisons of inference between models may enable us to posit new hypotheses for epidemiological study (e.g., vaccination of the elderly provides a protective effect among more susceptible and highly connected populations like children) (Fig AI in S1 Appendix).

Our model provides fine-scale, high coverage surveillance of ILI in the United States, allowing for a better understanding of influenza spatio-temporal patterns. Through an examination of significant group effects, we observed that South Atlantic states may experience longer and more acute seasons than other parts of the U.S during both seasonal and pandemic influenza scenarios and across ILI surveillance for children and adults. Our results also suggest that county-level spatial dependence and state effects explain a substantial part of the variation in epidemic intensity, while county-level spatial dependence and season effects best capture variation in epidemic duration. The explanatory power of county spatial dependence for surveillance models in both measures adds evidence to the importance of local mobility in the spatial spread and distribution of influenza disease burden [26, 56]. Moreover, we posit that state groupings explained variation in epidemic intensity because state-level policy recommendations and laws drive the probability for influenza infection and seeking of insured healthcare. For instance, influenza vaccination guidelines and access to free vaccinations are driven by local policy recommendations, and insurance policies are tied to state-level rules and regulations. Additional evidence for this hypothesis comes from our 2009 pandemic model where state effects also played a large role in explaining the variance in the data. On the other hand, variation in epidemic duration was better captured by season-level effects, and fixed effects that varied more between seasons than within them (e.g., influenza A/H3 and B circulation) were significant, similar to other studies [1]. We hypothesize that the duration of heightened ILI activity is more closely tied to population-level susceptibility and the identities of the predominantly circulating strains —factors that are likely to vary more across seasons than across space.

Our work uniquely captures factors of the measurement process, highlighting biases and disparities in healthcare-based influenza surveillance. We found that locations with greater poverty had lower influenza disease burden, in contrast to previous evidence for heightened rates of influenza-related hospitalizations, influenza-like illness, respiratory illness, neglected chronic diseases, and other measures of poor health among populations with greater material deprivation [43, 44, 47, 58–63]. Differences in socio-economic background may change recognition and therefore reporting of disease symptoms [46, 58]. Material deprivation and lack of social cohesion have also been implicated in lower rates of health care utilization for ILI, which would reduce the observation of influenza disease burden in our medical claims data among the poorest populations [44, 60]. When we artificially removed counties from our model (fixed-location sentinels) or subset our data into age groups, measurement factors associated with health care-seeking behavior more strongly explained the variation in epidemic intensity among the remaining observations (Fig 4, Fig AI in S1 Appendix). These two results together suggest that statistical inference from opportunistic data samples may avoid some types of reporting biases when the coverage or volume of data achieves a minimum threshold, in response to concerns posed in [14]. Increases to claims database coverage or care-seeking behavior may reduce reporting biases by increasing the representativeness of a given location’s sample, thus highlighting the importance of collecting and using metadata from opportunistic sources of epidemiological data.

Equipped with our model, we investigated the impact of surveillance system structure. We present the concept of a network of *sentinel locations*, in contrast to sentinel physicians or hospitals, which may be composed of administrative units (e.g., counties) that are chosen for either their representativeness of the larger population or their status as an outlier (e.g., match or failure to match locations in Fig 4, respectively). The ability for our model to estimate relatively accurate estimates of influenza burden across increasingly missing data suggests that routine sentinel surveillance in fixed locations may be more accurate for interpolating ILI disease burden among uncovered areas than surveillance across changing locations, even when fewer locations may be surveyed. Our framework enables sentinel counties to have flexible physician recruitment strategies, provided that county health departments can achieve target population coverage levels. Moreover, the improved performance of fixed-location surveillance systems is operationally ideal; as counties and physicians are retained as sentinels over long periods of time, we may expect the quality and consistency of reporting to improve. The accuracy of our surveillance model broke down at roughly 70% missingness among sentinels in fixed locations, which translates to fewer than 950 sentinel counties reporting data. While there are fewer sentinel counties than sentinel physicians in ILINet (approximately 2,000), we note that our county data represents aggregate reports from many healthcare providers. Indeed, the volume of visits captured by ILINet corresponded roughly to 5% of reporting counties in our medical claims data, and this level of missingness provided poor disease burden estimates for approximately 10-30% of counties in the best-case sentinel design (i.e., fixed-locations).

Our work contributes to our understanding of optimal population capture through surveillance by suggesting a framework that best maintains surveillance system design over multiple flu seasons [10–12]. Previous work acknowledges that spatial scales of aggregation alter statistical inference and statistically-identified drivers of disease distributions [64, 65]. Our aggregated state surveillance models adequately captured the high epidemic intensity risk among counties in the South Atlantic, similar to other studies of spatial scale [66], but they over-estimated epidemic intensity among low-risk states, thus suggesting that these types of surveillance models may be useful for public health preparedness but less optimal for the allocation of limited resources. Nevertheless, we observed that larger discrepancies between state- and county-level surveillance models were associated with greater within-state heterogeneity in disease burden, suggesting perhaps that the spatial aggregation of data may have minimal effects on epidemiological inference and policy-making if populations and socio-environmental determinants are relatively homogeneous within a given spatial unit (Fig Q in S1 Appendix). Overall, state surveillance models seemed more prone to over-estimate than under-estimate county-level disease burden, suggesting that inference from state surveillance data is best limited to populous counties in a given state (Fig P in S1 Appendix). Future work is needed to better understand surveillance-associated aggregation biases in order to expand the utility of aggregate scale surveillance data in local contexts.

Given the growing availability of health-associated big data in infectious disease surveillance [13, 67], we emphasize the importance of collecting relevant metadata on system coverage and reporting, while considering the ethical and privacy implications of using these data at fine spatial resolutions [14]. In the future, statistical surveillance modeling may become standard methodology to inform the choice of sentinel locations with non-traditional high-volume digital health data, improve the long-term design of disease surveillance systems, and enhance the development of syndromic surveillance in developing countries [68].

## Methods

### Medical claims data

Weekly visits for influenza-like illness (ILI) and any diagnosis from October 2002 to April 2010 were obtained from a records-level database of US medical claims managed by IMS Health and aggregated to three-digit patient US zipcode prefixes (zip3s), where ILI was defined with International Classification of Diseases, Ninth Revision (ICD-9) codes for: direct mention of influenza, fever combined with respiratory symptoms or febrile viral illness, or prescription of oseltamivir. Medical claims have been demonstrated to capture respiratory infections accurately and in near real-time [69, 70], and our specific dataset was validated to independent ILI surveillance data at multiple spatial scales and age groups and captures spatial dynamics of influenza spread in seasonal and pandemic scenarios [56, 71, 72]. Please see Section 1 in S1 Appendix for a statement on ethics and data access.

We also obtained database metadata from IMS Health on the percentage of reporting physicians and the estimated effective physician coverage by visit volume; these data were used to generate “measurement” predictors (Table 1). ILI reports and measurement factors at the zip3-level were redistributed to the county-level according to population weights derived from the 2010 US Census ZIP Code Tabulation Area (ZCTA) to county relationship file, assuming that ZCTAs that shared the first three digits belonged to the same zip3. These metadata indicated that our medical claims database represented roughly 24% of visits for any diagnosis from approximately 37% of all health care providers across 95% of U.S. counties during influenza season months, averaged over the years in our study period.

### Defining influenza disease burden

We performed the following data processing steps for each county-level time series of ILI per population (Section 7 in S1 Appendix): i) Fit a LOESS curve to non-flu period weeks (flu period defined as November through March each year) to capture moderate-scale time trends (span = 0.4, degree = 2); ii) Subtract LOESS predictions from original data to detrend the entire time series; iii) Fit a linear regression model with annual harmonic terms and a time trend to non-flu period weeks [16]; iv) Counties were defined to have an “epidemic” in a given flu season if at least two consecutive weeks of detrended ILI observations exceeded the ILI epidemic threshold during the flu period (i.e., epidemic period) [73]. The epidemic period was the maximum length consecutive period where detrended ILI exceeded the epidemic threshold during the flu period. The epidemic threshold was the upper bound of the 95% confidence interval for the linear model prediction. Counties with a greater number of consecutive weeks above the epidemic threshold during the non-flu period than during the flu period were removed from the analysis; v) Disease burden metrics were calculated for counties with epidemics.

Two measures of influenza disease burden were defined for each county. For a given season and county: We define attack rate as the sum of population-normalized and detrended ILI during the epidemic period found above (and shifted by one to accommodate the likelihood distribution). Our *epidemic intensity* measure is defined as the standardized ratio of this attack rate and the expected attack rate. The expected attack rate is calculated as the population-weighted mean of the observed attack rates, and is a model offset described under ‘Model structure’. *Epidemic duration* was defined as the number of weeks in the epidemic period and counties without epidemics were assigned the value zero.

Models and data were processed separately for the 2009 H1N1 pandemic season and for state-level epidemic intensity (details in Sections 2.5 and S2.6 in S1 Appendix respectively).

### Predictor data collection and variable selection

Quantifiable proxies were identified for each hypothesis found in the literature, and these mechanistic predictors were collected from probability-sampled or gridded, publicly available sources and collected or aggregated to the smallest available spatial unit among US counties, states, and Department of Health and Human Services (HHS) regions for each year or flu season in the study period, as appropriate (Table 1, Section 6 in S1 Appendix).

We selected one predictor to represent each hypothesis according to the following criteria, in order: i) Select for the finest spatial resolution; ii) Select for the greatest temporal coverage for years in the study period; iii) Select for limited multicollinearity with predictors representing the other hypotheses, as indicated by the magnitude of Spearman rank cross-correlation coefficients between predictor pairs. We also compared the results of single predictor models and our final multi-predictor models as another check of multicollinearity (Section 6 in S1 Appendix). For the modeling analysis, if a predictor had missing data at all locations for an entire year, data from the subsequent or closest other survey year were replicated to fill in that year. If a predictor data source was available only at the state or region-level, all inclusive counties were assigned the corresponding state or region-level predictor value (e.g., assign estimated percentage of flu vaccination coverage for state of California to all counties in California). Predictors were centered and standardized prior to all exploratory analyses and modeling, as appropriate. Interaction terms comprised the product of their component centered and standardized predictors. Data cleaning and exploratory data analysis were conducted primarily in R [74]. Final model predictors are described below, and our hypotheses for each predictor are described in Table 1. All cleaned predictor data are available upon request.

#### Environmental data

Daily specific humidity data on a 2m grid were collected from the National Oceanic and Atmospheric Administration (NOAA) North American Regional Reanalysis (NARR), provided by the NOAA/OAR/ESRL PSD, Boulder, Colorado, USA, from their website at http://www.esrl.noaa.gov/psd/. Values were assigned to the grid point nearest to the county centroid.

Readings of fine particulate matter, defined as pollutants with aerodynamic diameter less than 2.5 micrometers, were collected from the CDC WONDER database at the county and daily scales from their website at https://wonder.cdc.gov/.

#### Social contact and population data

Annual total and age-specific population data were taken from the intercensal population estimates and land area and number of housing units were reported during the 2000 and 2010 Census; both datasets were available at the county scale from the U.S. Census Bureau. These data were used to calculate proportion of total population that are children (5-19 years old) and adults (20-69 years old), population density by land area, and estimated average household size.

#### Flu-specific data

Annual flu vaccination rates for toddlers (19-35 months old) and the elderly (≥ 65 years old) were estimated at the state-level from the Centers for Disease Control and Prevention (CDC) National Immunization Survey and Behavioral Risk Factor Surveillance System, respectively. Annual proportion of A-typed flu samples subtyped as H3 and annual proportion of confirmed flu samples typed as B across U.S. Department of Health and Human Services (HHS) regions were collected by WHO/NREVSS Collaborating Labs and available at the CDC FluView website at http://www.cdc.gov/flu/weekly/fluviewinteractive.htm.

#### Prior immunity

For a given county, a proxy for prior immunity was derived from the following data: 1) the previous flu season’s total population epidemic intensity; the proportion of positive flu strains identified as A/H3, A/H1, and B in the broader HHS region during 2) the previous flu season and 3) the current flu season; 4) the most prominently circulating flu strain for each category (A/H3, A/H1, or B) for each flu season; 5) antigenic clusters for A/H3 and A/H1 strains as identified in [75, 76]; and 6) Victoria- or Yamagata-like lineages for B strains as noted in [77]. Data for items 1-3 are described above in “Defining influenza disease burden” and “Flu-specific data.” We obtained the antigenic characterizations for circulating strains (item 4) from CDC influenza season summaries, which are available at https://www.cdc.gov/flu/weekly/pastreports.htm.

Using these data, we calculated a proxy of prior immunity that captures “the proportion of individuals infected in the previous flu season that would have protection during the current flu season, accounting for the distribution of circulating flu strains” (Section 7 in S1 Appendix). For each flu category among A/H3, A/H1, and B, we calculated the product of the previous and cxurrent year’s proportion of total circulation and a binary value to indicate if previous and current strains were from the same antigenic cluster or lineage (1 = same cluster/lineage, 0 = different cluster/lineage). For a given county, these products were summed across A/H3, A/H1, and B, and multiplied by the previous year’s epidemic intensity.

#### Socioeconomic and access to care data

Annual data on number of hospitals were obtained at the county-level from the Health Resources and Services Administration (HRSA) Area Health Resources Files (AHRF). County-level data on proportion of households with a single person were obtained from five-year averages of American Community Survey (ACS) estimates, which were available starting in 2005. Annual estimates on proportion of the population in poverty was obtained at the county-level from the model-based Small Area Income and Poverty Estimates (SAIPE). Annual estimates on proportion of the population with health insurance was obtained at the county-level from the model-based Small Area Health Insurance Estimates (SAHIE). SAIPE and SAHIE are both products of the U.S. Census Bureau that were derived from the Current Population Survey or ACS.

#### Medical claims measurement factors

IMS Health provided us with weekly aggregated data on visits for any diagnosis by age group and location. Care-seeking behavior was defined as the total visits per population size from November through April of a given flu season. Claims database coverage was the estimated physician coverage among all physicians registered by the American Medical Association in the IMS Health medical claims database.

### Model structure

We present the most common version of our model structure here. The generic model for county-year observations (for *i* counties and *t* years) of influenza disease burden **y**_*t*_ is:

$$\begin{array}{c} y_{t}|\mu_{t},\tau ∼f\left(y_{t}|\mu_{t},\tau \right) \end{array}$$ (1)

where **y**_*t*_ = (*y*_1*t*_, …, *y*_*nt*_)′ denotes the vector of *i* = 1, …, *n* county observations across *t* = 1, …, *T* years included in the model (Eq 1). We modeled the mean of the observed disease burden magnitude (***μ***_*t*_), where *f*(**y**_*t*_|***μ***_*t*_, *τ*) is the distribution of the likelihood of the disease burden data, parameterized with mean ***μ***_*t*_ = (*μ*_1*t*_, …, *μ*_*nt*_)′ and precision *τ* (where precision is the inverse of variance), as appropriate to the likelihood distribution.

The proposed determinants of disease burden were modeled as:

$$\begin{array}{c} g\left(\mu_{it}\right)=E_{it}+\alpha +\sum_{p=1}^{m}X_{itp}\beta_{p}+\gamma_{i}+\zeta_{j[i]}+\eta_{k[i]}+\nu_{t}+\phi_{i}+\epsilon_{it} \end{array}$$ (2)

where *g*(.) is the link function, *α* is the intercept, there are *m* socio-environmental and measurement predictors (i.e., **X**_*t*1_, …, **X**_*tm*_), where **X**_*t*1_ = (*X*_1*t*1_, …, *X*_*nt*1_)′, and *E*_*it*_ is an offset of the expected disease burden, such that Eq 2 models the relative risk of disease (*μ*_*it*_/*E*_*it*_) in county *i*, common in disease mapping [78–80]. Group terms at the county, state *j*, region *k*, and season *t* levels (*γ*_*i*_, *ζ*_*j*[*i*]_, *η*_*k*[*i*]_, *ν*_*t*_, respectively) and the error term (*ϵ*_*it*_) are independent and identically distributed (*iid*).

Geographical proximity appears to increase the synchrony of flu epidemic timing [81, 82], while connectivity between cities has been linked with spatial spread in the context of commuting and longer distance travel [83–86]. We modeled county spatial dependence *ϕ*_*i*_ with an intrinsic conditional autoregressive (ICAR) model, which smooths model predictions by borrowing information from neighbors [87]:

$$\begin{array}{c} \phi_{i}|\phi_{j,-i},\tau_{\phi }∼\text{Normal}\left(\frac{1}{\xi_{i}}\sum_{i∼j}\phi_{j},\frac{1}{\xi_{i}\tau_{\phi }}\right), \end{array}$$ (3)

where *ξ*_*i*_ represents the number of neighbors for node *i*, ***ϕ***_***j***,−***i***_ represents the neighborhood of node *i*, which is composed of neighboring nodes *j* (neighbors denoted *i* ∼ *j*). The precision parameter is *τ*_*ϕ*_ (Eq 3).

### Statistical analysis

The goals of our modeling approach were to i) estimate the contribution of each predictor to influenza disease burden, ii) predict disease burden in locations with missing data, and iii) improve mapping of influenza disease burden. We performed approximate Bayesian inference using Integrated Nested Laplace Approximations (INLA) with the R-INLA package (www.r-inla.org) [88, 89]. INLA has demonstrated computational efficiency for latent Gaussian models, produced similar estimates for fixed parameters as established implementations of Markov Chain Monte Carlo (MCMC) methods for Bayesian inference, and been applied to disease mapping and spatial ecology questions [90–94].

Log epidemic intensity was modeled with a normal distribution, and log epidemic duration was modeled with a normal distribution without the offset term in Eq 2. Consequently, we note that all epidemic intensity models examine the relative risk of disease burden, while epidemic duration models examine the duration in weeks. Multi-season models included all terms in Eq 2. Model coefficients were interpreted as statistically significant if the 95% credible interval for a parameter’s posterior distribution failed to include zero.

### Model assessment and validation

To assess model fit, we examined scatterplots and Pearson’s cross-correlation coefficients between observed and fitted values for the epidemic intensity and epidemic duration total population surveillance models. The epidemic intensity model fit the data well and the Pearson’s cross-correlation coefficient between the observed and fitted mean relative epidemic intensity was *R* = 0.86 (Section 2 in S1 Appendix). The epidemic duration model fit relatively well, and the Pearson’s cross-correlation coefficient between the observed and predicted mean number of epidemic weeks was *R* = 0.94 (Section 4 in S1 Appendix).

We also examined scatterplots of standardized residuals and fitted values; standardized residuals were defined as $\left(y-\mu_{\hat{y}}\right)/\sigma_{\hat{y}}$, where $\mu_{\hat{y}}$ is the fitted value posterior mean and $\sigma_{\hat{y}}$ is the fitted value standard deviation. Residual plots for the epidemic intensity and duration models may be found in Sections 2 and 4 in S1 Appendix, respectively.

For each disease burden measure, we compared models with no spatial dependence, county-level dependence only, state-level dependence only, and both county and state-level dependence. The goal of the county-level dependence was to capture local population flows, while state-level dependence attempted to capture state-level flight passenger flows (details in Section 2 in S1 Appendix). We determined that models with only county-level spatial neighborhood structure best fit the data after examining the Deviance Information Criteria (DIC) values and spatial dependence coefficients of the four model structures, further supporting evidence in [56]. County-level spatial structure was subsequently used in all final model combinations. We report results from models with county-level dependence only.

We assessed the contribution of each set of group effects (i.e., season, region, state, county, county spatial dependence, observation error) to model fit by comparing the mean precision estimates for the terms, where precision is the inverse of variance. Effects with a smaller precision captured a greater magnitude of variability in the data.

We examined the added value of county-level information relative to state-level information by comparing the aggregation bias between county and state surveillance models. Here, we defined aggregation bias as the difference between fitted log epidemic intensity from state and county surveillance models. Positive values mean that the state model overestimates risk relative to the county model, and vice versa.

For model validation, we compared model fitted values for epidemic intensity with CDC ILI and laboratory surveillance data, which are derived from approximately 2,000 ILI-reporting sentinel physicians and 100,000-200,000 respiratory specimens annually (details in Section 2 in S1 Appendix). We assessed model robustness through additional cross-validation and out-of-sample validation analyses; the total population epidemic intensity model was refit where 20%, 40%, 60%, 80%, 90%, 95%, and 97.5% of all county observations were randomly replaced with NAs (*sentinels in fixed locations*), and where 20%, 40%, 60% and 80% of model observations were stratified by season and randomly replaced with NAs (*sentinels in moving locations*). We also refit three models where one, three, and five of seven flu seasons were randomly chosen and completely replaced with NAs (*inclusion of historical data*). To account for variability due to random chance, models were replicated ten times each with different random seeds. For each sequence of missingness, we performed out-of-sample validation by comparing the mean fitted values to the true observed values for all data that were randomly removed across seasons and replicates (Section 3.4 in S1 Appendix). We then compared the magnitude and significance of socio-environmental and measurement drivers, and the posterior distributions of county-season fitted values. Fitted value distributions were noted as significantly different (i.e., values did not match) if the interquartile ranges for two fitted values failed to overlap with each other (Section 3.2 in S1 Appendix). The results described in “Sentinel surveillance design” use methods identical to this analysis and may be interpreted additionally as model sensitivity and robustness.

### Availability of model codes and outputs

Model estimates of disease burden, summary statistics for predictors, and their associated model codes are openly available on GitHub at https://github.com/bansallab/optimize-flu-surveillance. All processed predictor data are available upon request.

## Supporting information

S1 Appendix Supplemental figures for the surveillance models, data validation, sensitivity analyses, and model predictors. This content includes Sections 1 to 7, Tables A to F, and Figures A to AR.
(PDF)
