# Supplementary material for: Deploying digital health data to optimize influenza surveillance at national and local scales
Source: PLoS Comput Biol. 2018 Mar 7;14(3):e1006020. doi: 10.1371/journal.pcbi.1006020 (PMC5858836; doi:10.1371/journal.pcbi.1006020)
Supplement: S1 Appendix — This content includes Sections 1 to 7, Tables A to F, and Figures A to AR. (PDF) [file pcbi.1006020.s001.pdf]

Appendix: Deploying digital health data to optimize influenza surveillance at national and local scales

Contents

1 **Ethics and data access** 3

2 **Epidemic intensity surveillance models** 3

2.1 Surveillance model and spatial predictors . . . 3
2.2 Influenza surveillance and spatial patterns . . . 4
2.3 Selection for spatial dependence terms . . . 4
2.4 Validation to CDC surveillance data . . . 5
2.5 2009 pandemic surveillance model and spatial predictors . . . 5
2.6 State seasonal intensity surveillance model . . . 9

3 **Sentinel surveillance design** 11

3.1 Comparison of county and state spatial units of analysis . . . 11
3.2 Thresholds to identify match . . . 11
3.3 Sentinels in moving locations . . . 11
3.4 Out-of-sample validation . . . 11

4 **Epidemic duration surveillance models** 17

4.1 Surveillance model and spatial predictors . . . 17
4.2 Influenza surveillance and spatial patterns . . . 17
4.3 Comparison of epidemic duration and epidemic intensity . . . 20

5 **Epidemic intensity surveillance models for children and adults** 20

5.1 Surveillance model and spatial predictors . . . 20
5.2 Influenza surveillance and spatial patterns . . . 24

6 **Model predictors** 24

6.1 Checks for multicollinearity . . . 24
6.2 Medical claims coverage . . . 24

7 **Data processing** 27

7.1 Defining influenza disease burden . . . 27
7.2 Prior immunity tables . . . 27
7.3 Comparison to alternative data processing steps . . . 29

Group effect	Mean of the precision estimate
Season	49.92
Region	15.29
State	5.08
County	21,951.48
County spatial dependence	1.63
Observation error	18,175.80

Table A. Comparison of the mean precision estimates for group effects in the intensity models, where precision is the inverse of the variance.

1 Ethics and data access

Patient records and information in the medical claims dataset were anonymized, de-identified, and aggregated by IMS Health, a data analytics firm that packages data that is routinely collected for health insurance purposes for commercial use. While the database is not accessible online, interested researchers should refer to the IMS Health (now IQVIA) website: https://www.iqvia.com/. All analyses were performed with aggregated time series data for influenza-like illness rather than patient-level information. Similar to other epidemiological analyses of administrative insurance data, no institutional review board approval was sought.

2 Epidemic intensity surveillance models

2.1 Surveillance model and spatial predictors

We assessed model diagnostics and surveillance outputs for the total population epidemic intensity model(Figure A, Figure B, Figure E). We examined the mean precision estimates for the group effects —season, region, state, county, county spatial dependence, observation error —to identify which effects explained most of the variability across observations (Table A).

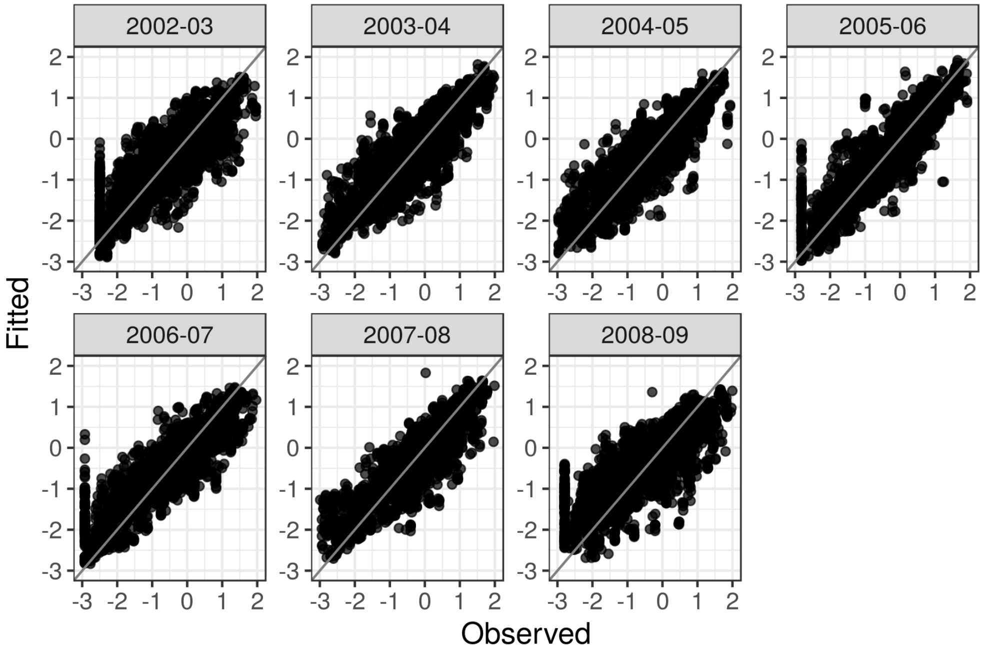

Figure A. Observed vs. fitted means for log epidemic intensity (Pearson's $R = 0.86$). The 45 degree line is in grey.

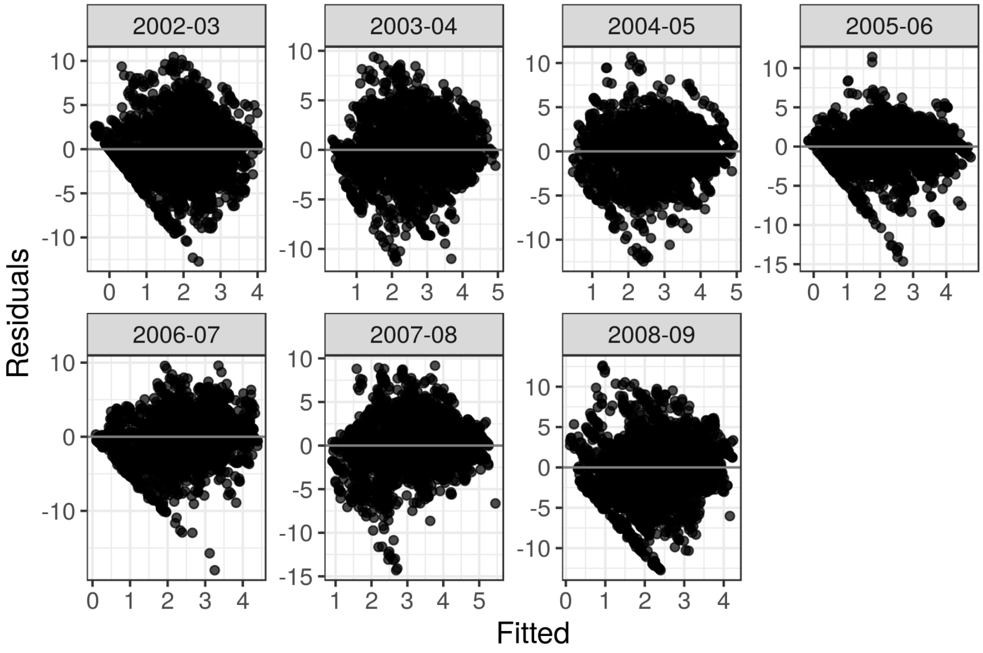

Figure B. Residuals vs. fitted means for the total population log epidemic intensity.

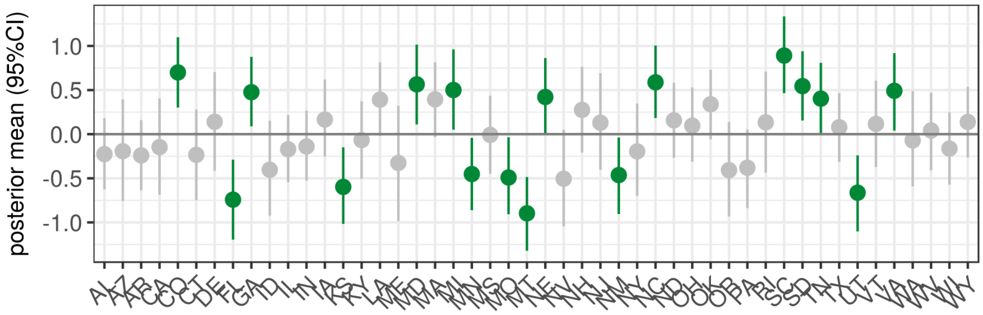

Figure C. 95% credible intervals for state group effects for the total population epidemic intensity model.

2.2 Influenza surveillance and spatial patterns

We examined the spatial and temporal patterns for the epidemic intensity model (Figure 2, Figure C, Figure D, Figure E).

2.3 Selection for spatial dependence terms

To determine county-level spatial neighbors, we started with the 2010 U.S. Census Bureau 500k resolution county shapefile, and connected abutting counties that were separated by bodies of water. We then used the clean shapefile to identify neighbors as counties that shared borders.

To define state-level spatial neighbors, monthly air travel passenger flows were collected from the Bureau of Transportation Statistics T-100 Domestic Market (U.S. Carriers) table from their website at http://www.transtats.bts.gov/. Airport flows were aggregated to the state-level and states were neighbors if passengers traveled between them from November 2007 through April 2008.

Models with the county only or county and state-level spatial dependence terms had the lowest DIC (Table B). As none of the spatially-structured state-level coefficients were significantly different from zero, we chose to use models with county-level dependence only.

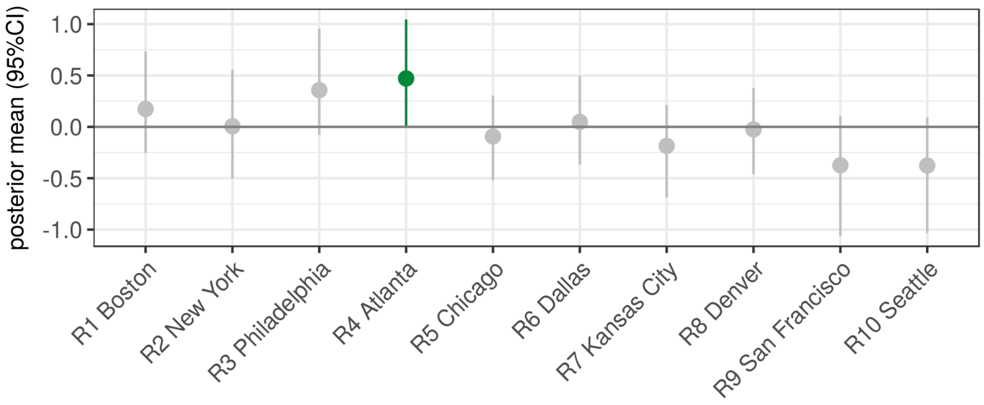

Figure D. 95% credible intervals for HHS region group effects for the total population epidemic intensity model.

Spatial dependence structure	DIC
None (no ϕ terms)	40,932
County only (bordering neighbors, ϕ_i)	40,070
State only (flight passenger flows, ϕ_j)	40,933
County and state together (ϕ_i and ϕ_j)	40,070

Table B. Comparison of total epidemic intensity models with different spatial dependence structures and random effects according to Deviance Information Criterion (DIC).

2.4 Validation to CDC surveillance data

We collected a) the percentage of ILI out of all patient visits among the total population, and child and adult populations as reported by CDC's ILINet, and b) the percentage of positive influenza laboratory confirmations as reported by CDC laboratory surveillance. We note that child and adult ILI percentage was calculated with a denominator of patient visits across all age groups due to limited data availability. Both CDC surveillance systems were reported at the HHS region level and aggregated cumulatively for each flu season in our study period. We then examined scatterplots and Pearson cross-correlation coefficients (double-sided test where H_o = no difference) between the mean model fits for epidemic intensity (where we took the mean across all counties in a given HHS region) and each CDC surveillance dataset (Figure G, Figure H, Figure I, Figure J). Results were similar to those reported for medical claims ILI intensity in Viboud et al. 2014[1].

2.5 2009 pandemic surveillance model and spatial predictors

We developed a separate model to characterize the spatial and temporal patterns of the fall wave of the 2009 H1N1 pandemic in the United States (Figure K, Figure L, Figure N). Using medical claims data from October 2002 to April 2010, we calculated epidemic intensity for the fall wave by adjusting the methodology we used to define disease burden; the flu period was defined as August through January instead of November through April during the pandemic year. In addition, we modified some of the predictors included in our model. Specific humidity and pollution measurements were averaged across the modified flu period from August through January as opposed to the original flu period of November through April. Toddler and elderly vaccination coverage were removed as predictors and replaced with estimates of child (6 months through 17 years) and adult (18 years and above) monovalent 2009 pandemic vaccination coverage; these state-level estimates were derived jointly from the CDC's National 2009 H1N1 Flu Survey (NHFS) and the Behavioral Risk Factor Surveillance System (BRFSS)[2]. Prior immunity was calculated as the sum of the adult and elderly proportions of the population multiplied by the reported proportion of pre-2009 serum with cross-reactive antibodies in those age groups[3]. Influenza circulation predictors were removed from the model because they should not have applied to the pandemic season. All other predictors were the same as

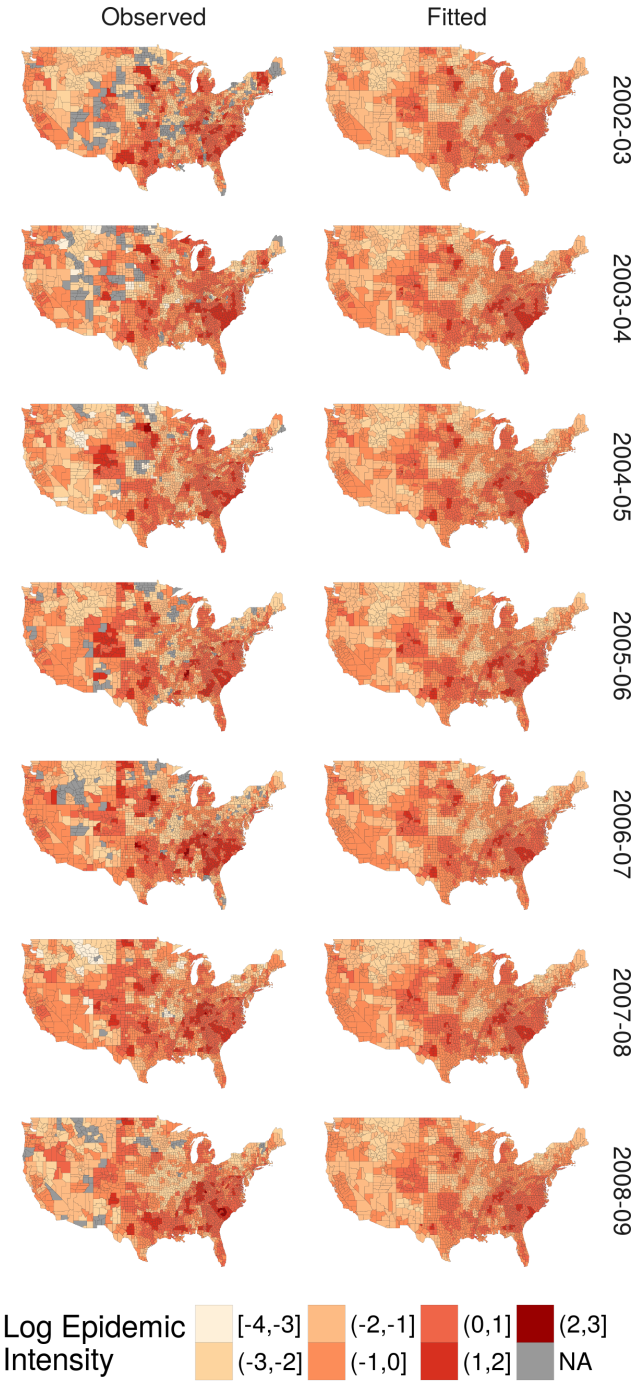

Figure E. Continental U.S. county maps for observed (left) and fitted (right) log epidemic intensity in seasons from 2002-03 through 2008-09.

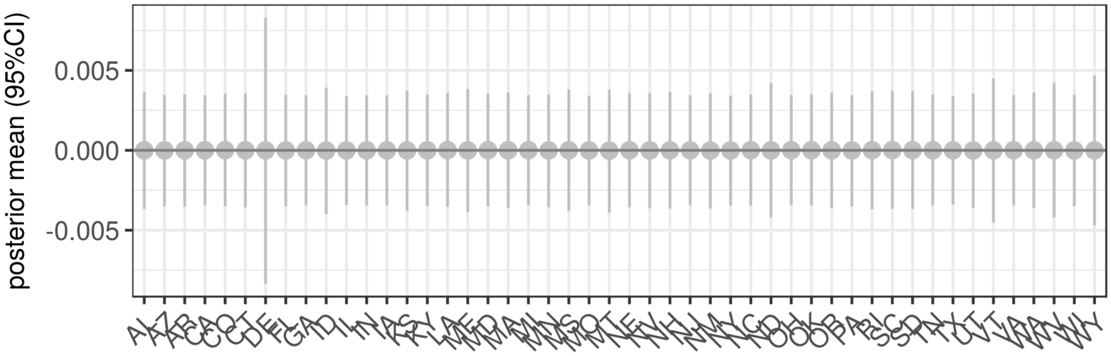

Figure F. 95% credible intervals for the state-level spatially structured coefficients when modeling intensity with state-level spatial dependence (ϕ_j). None of the spatially structured state coefficient distribution were significant.

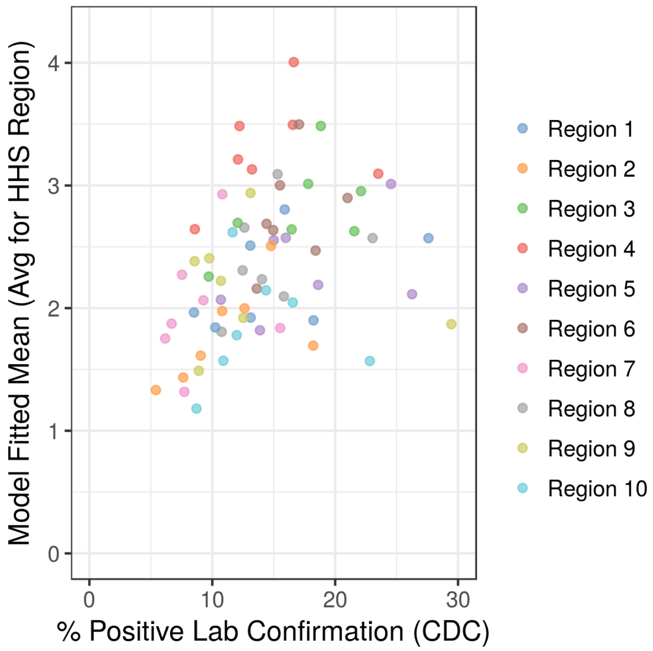

Figure G. Mean epidemic intensity model fit averaged across counties in a given HHS region vs. percentage of positive influenza laboratory confirmations in a given HHS region and flu season. The Pearson cross-correlation coefficient was 0.35 with a p-value of 0.003 for a double-sided hypothesis test.

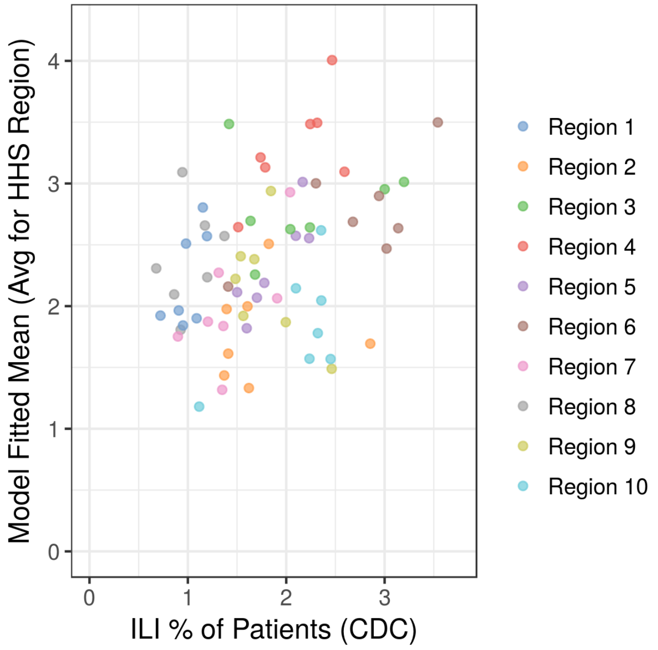

Figure H. Mean epidemic intensity model fit averaged across counties in a given HHS region vs. cumulative percentage of ILI visits in a given HHS region for all age groups. The Pearson cross-correlation coefficient was 0.38 with a p-value of 0.001 for a double-sided hypothesis test.

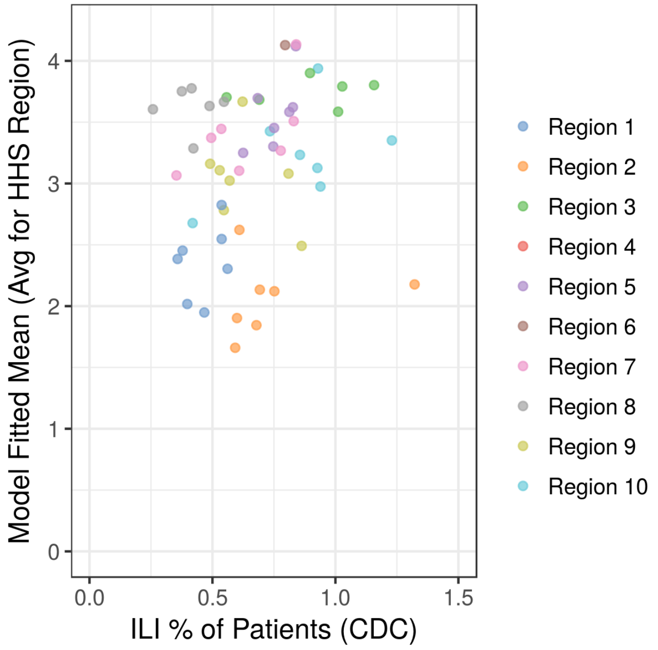

Figure I. Mean epidemic intensity model fit averaged across counties in a given HHS region vs. cumulative percentage of ILI visits in a given HHS region for children. The Pearson cross-correlation coefficient was 0.42 with a p-value of 0.0002 for a double-sided hypothesis test.

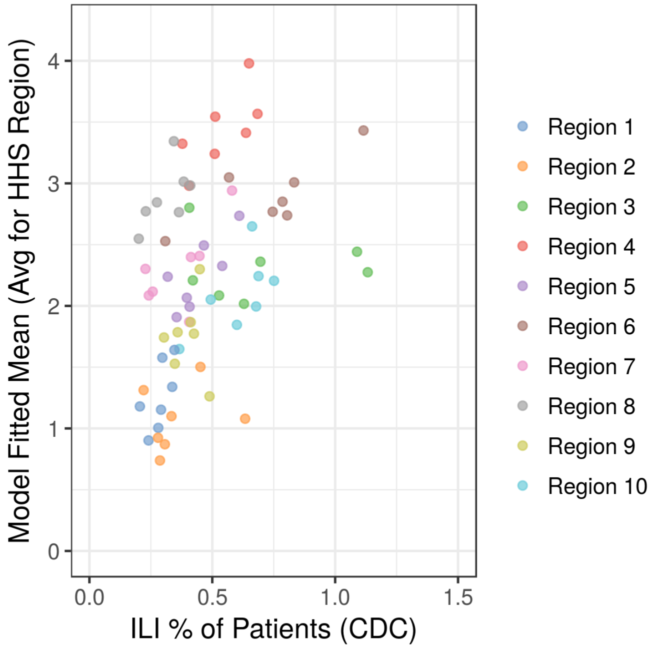

Figure J. Mean epidemic intensity model fit averaged across counties in a given HHS region vs. cumulative percentage of ILI visits in a given HHS region for adults. The Pearson cross-correlation coefficient was 0.42 with a p-value of 0.0003 for a double-sided hypothesis test.

Group effect	Mean of the precision estimate
State	3.66
County (Observation error)	5.65

Table C. Comparison of the mean precision estimates for group effects in the pandemic model, where precision is the inverse of the variance.

those used for the 2008-2009 influenza season. We excluded the county-level spatial dependence term because the model fit was better without it.

We examined the mean precision estimates for the group effects —state, county (same as observation error in this model) —to identify which effects explained most of the variability across observations in the pandemic model (Table C). State group effects (Figure M) then county effects explained the greatest amount of variability across pandemic model observations.

2.6 State seasonal intensity surveillance model

We developed a state-level epidemic intensity model to compare to our county-level epidemic intensity model (Figure O). All spatial predictors included in the county surveillance model were collected or aggregated to the state spatial unit of analysis and the expected epidemic intensity was recalculated from state-level epidemic intensity observations. The state surveillance model had the same structure as the county surveillance model but for the exclusion of the county group effects. We also excluded the state-level spatial dependence term because the model fit was better without it.

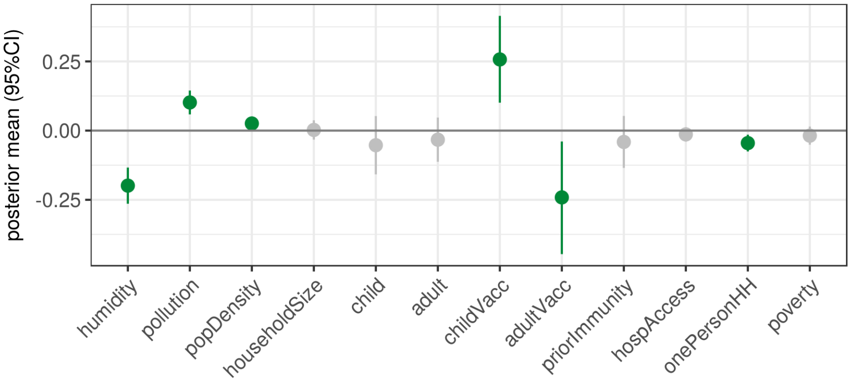

Figure K. For the pandemic intensity model, these are the means and 95% credible intervals for the posterior distributions of the socio-environmental coefficients.

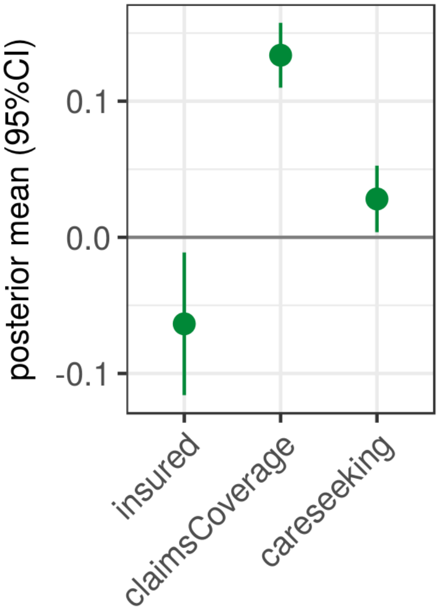

Figure L. For the pandemic intensity model, these are the means and 95% credible intervals for the posterior distributions of the measurement coefficients.

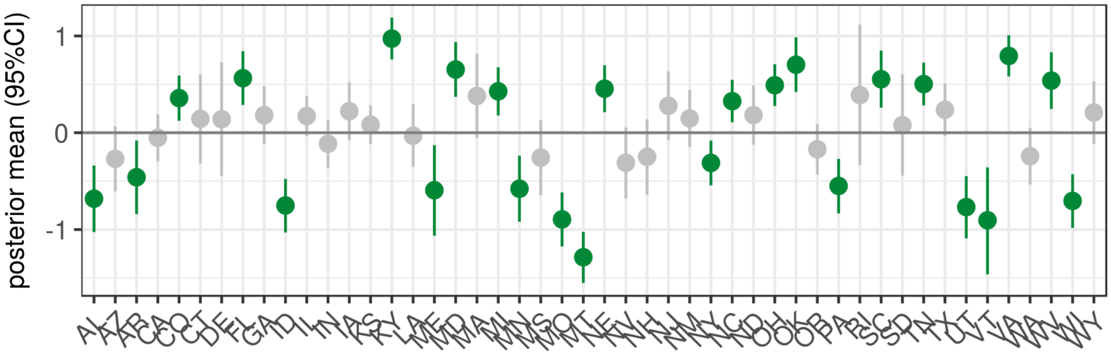

Figure M. 95% credible intervals for state group effects for the pandemic intensity model.

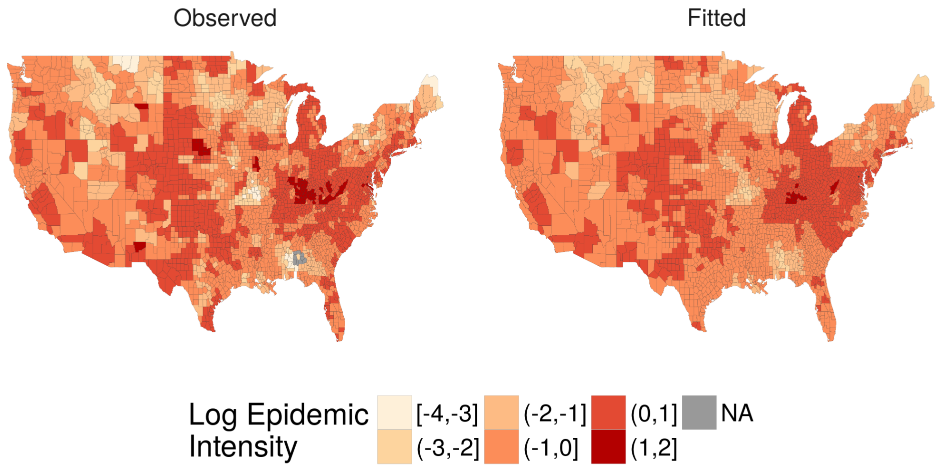

Figure N. Continental U.S. county map for observed (left) and fitted (right) log epidemic intensity during the fall wave of the 2009 H1N1 pandemic.

3 Sentinel surveillance design

3.1 Comparison of county and state spatial units of analysis

We compared the difference between the fitted value outputs of the state and county surveillance models to examine the added value of county-level information (Figure P). We also note that states with a larger magnitude of aggregation bias (error) have greater within-state heterogeneity in epidemic intensity (Pearson's correlation coefficient is 0.67, p-value $<$ 2E-16, $H_a =$ Linear correlation is statistically different from 0) (Figure Q). Absolute magnitude of aggregation bias was calculated as the sum of the absolute value of the error for all counties in a given state, where error is defined as the difference between the fitted log epidemic intensity for the state and county surveillance models. Within-state heterogeneity was indicated with the variance of the posterior means for fitted epidemic intensity derived from the county models across all counties in a given state.

3.2 Thresholds to identify match

We examined the empirical distribution of matched replicates (interquartile ranges of the fitted posterior distributions between the complete model and model with missingness do not overlap) by flu season for each level of missingness among sentinels in fixed locations (Figure R). At the lowest level of missing data examined (i.e., 80% of counties reporting data), nearly 90% of counties had matches in 9 of 10 replicates. Using this information, we set the threshold for match at 90% of county-season observations.

3.3 Sentinels in moving locations

We report the results for *sentinels in moving locations* (Figure S, Figure T) and *inclusion of historical data* (Figure U, Figure V) as described in Fig 4.

3.4 Out-of-sample validation

Models performed for the sentinel surveillance design analysis were also used in an out-of-sample validation procedure. For each sequence of missingness, we compared the fitted values to the known observation for all observation-replicates that were excluded from the model fitting procedure (Figure W, Figure X, Figure Y). These figures are pooled across different levels of missing data for a given sequence of missingness (e.g., fixed-location sentinels). If there is a strong linear relationship between the 'observed and excluded' values and the fitted values, the model performs well on observations that are 'out-of-sample.'

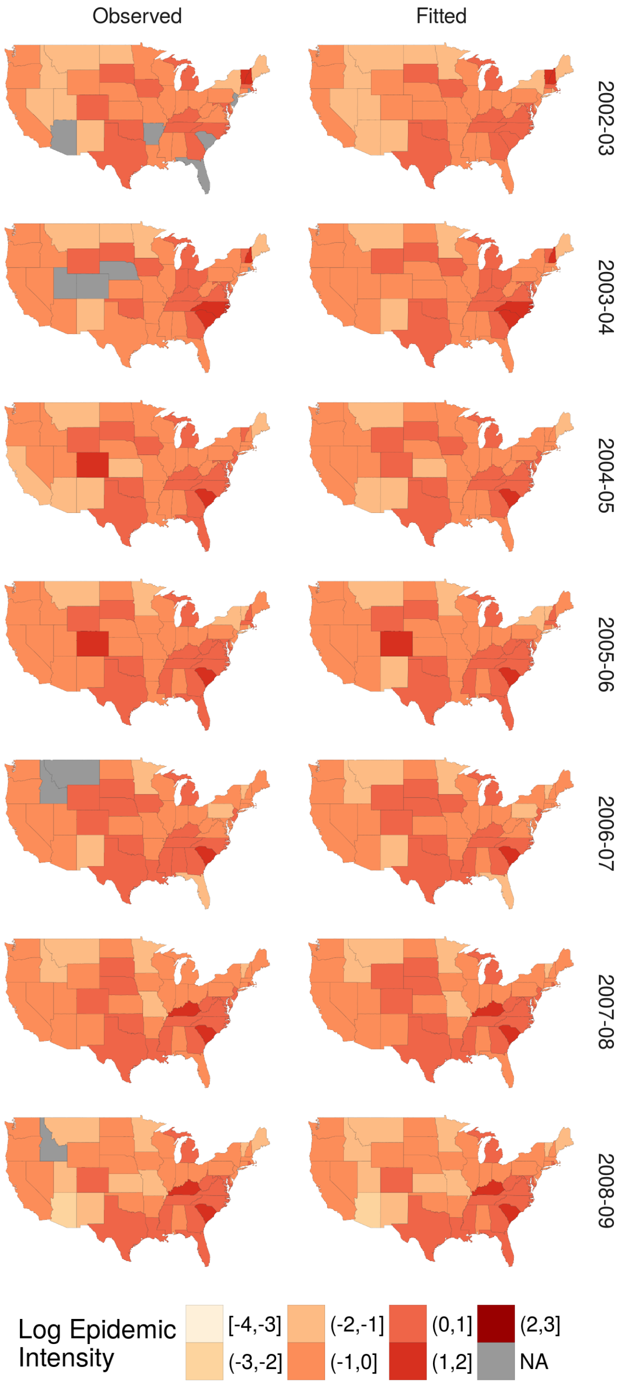

Figure O. Continental U.S. state maps for observed (left) and fitted (right) log epidemic intensity in seasons from 2002-03 through 2008-09.

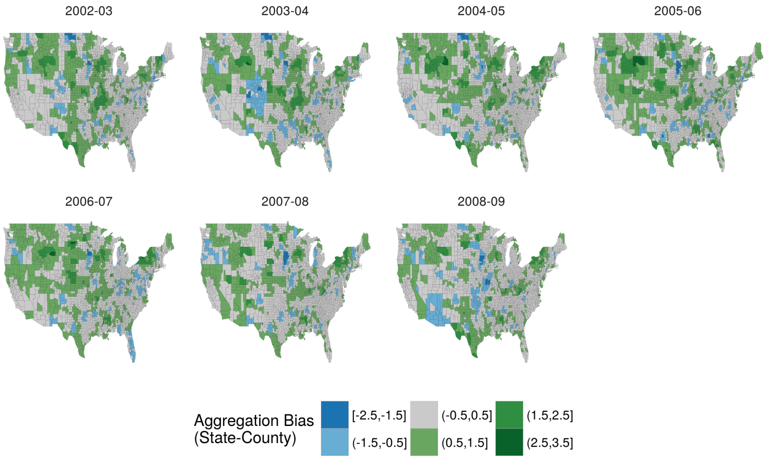

Figure P. Aggregation bias between county and state epidemic intensity surveillance models for all seasons in the study period, where aggregation bias is defined as the difference between fitted values for county and state log epidemic intensity. Negative error (blue) indicates that the state-level surveillance model underestimated risk relative to the county-level surveillance model, and vice versa.

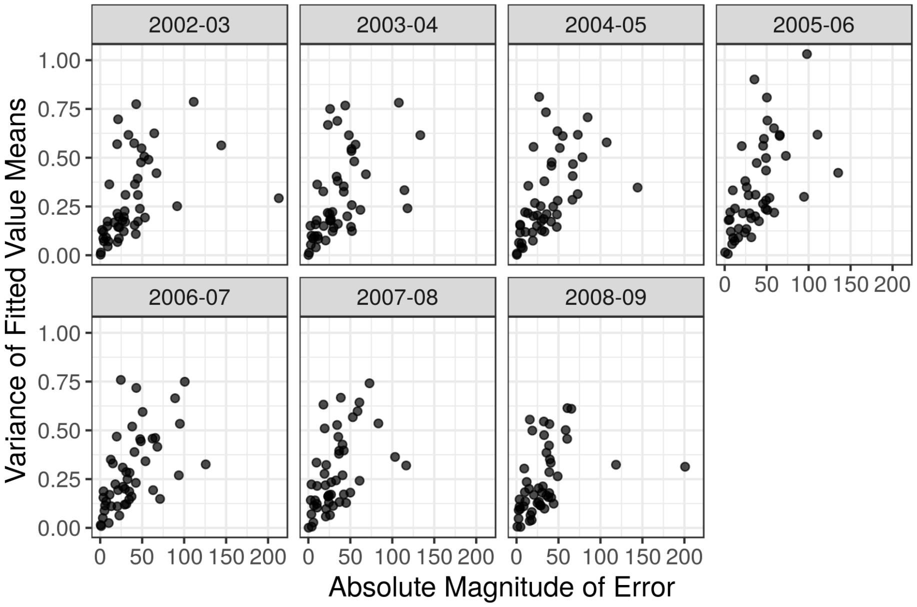

Figure Q. Scatterplot demonstrating that within-state heterogeneity of epidemic intensity increases as aggregation bias increases, where each data point represents a single state-season combination.

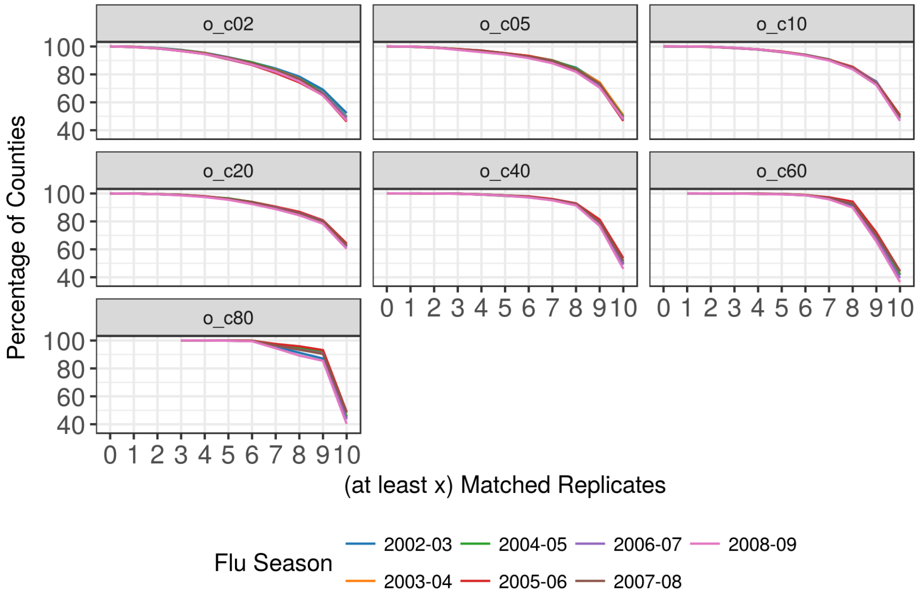

Figure R. Line plot showing the percentage of counties with at least *x* matched replicates by season for fixed-location sentinel models. Each panel displays results from a different level of missingness (2.5% of counties reporting data in the top left, to 80% of counties reporting data in the bottom left).

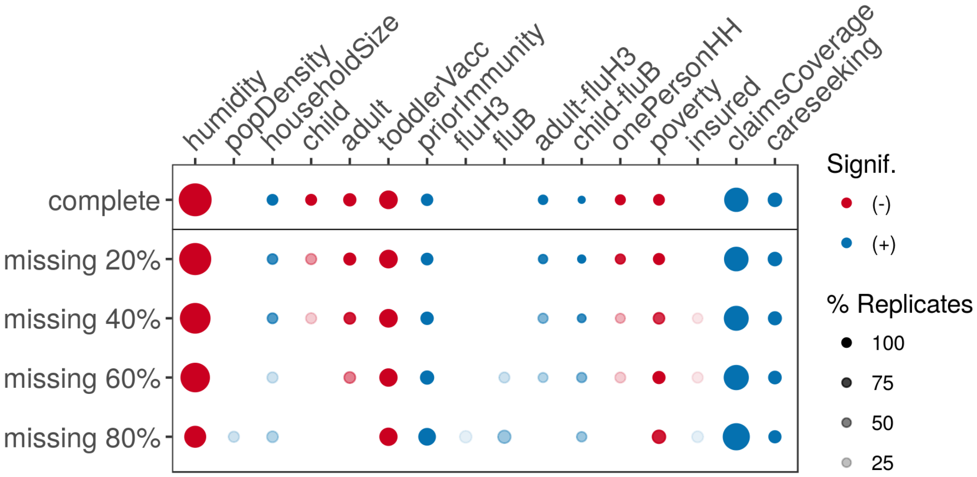

Figure S. Diagram indicating changes to model inference as fewer moving-location sentinels reported data. Predictors with no significant effect across the sequence of models were removed for viewing ease, and absence of a dot means the effect was not significant across any replicates.

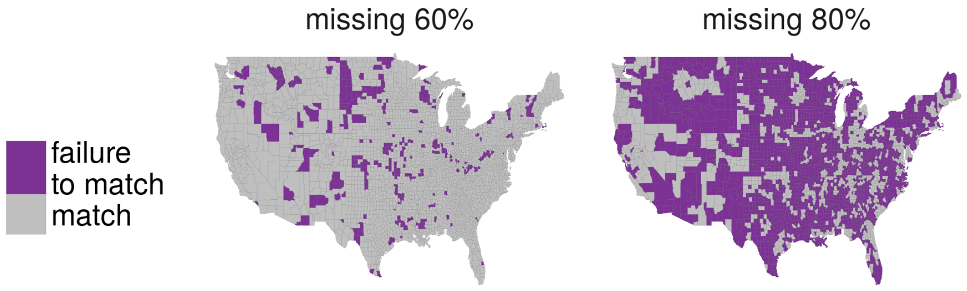

Figure T. Map of model prediction match between the complete model and the 60% and 80% missing levels for moving-location sentinels. Failure to match means that the interquartile ranges for two posterior distributions failed to overlap with each other in at least one replicate.

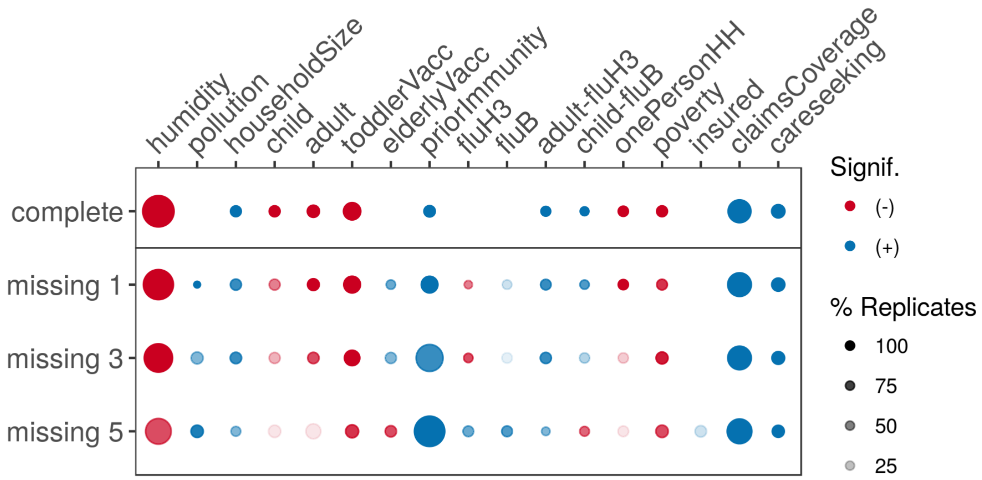

Figure U. Diagram indicating changes to model inference as historical seasons were randomly removed from the model. Predictors with no significant effect across the sequence of models were removed for viewing ease, and absence of a dot means the effect was not significant across any replicates.

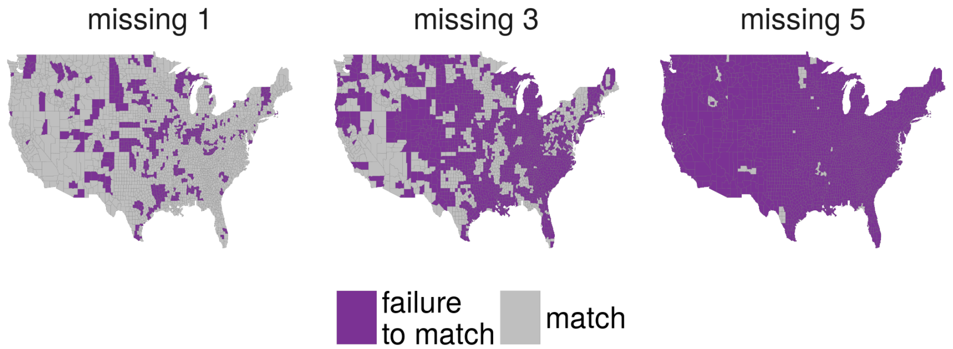

Figure V. Map of model prediction match between the complete model and models missing one, three, or five historical flu seasons. Failure to match means that the interquartile ranges for two posterior distributions failed to overlap with each other in at least one replicate.

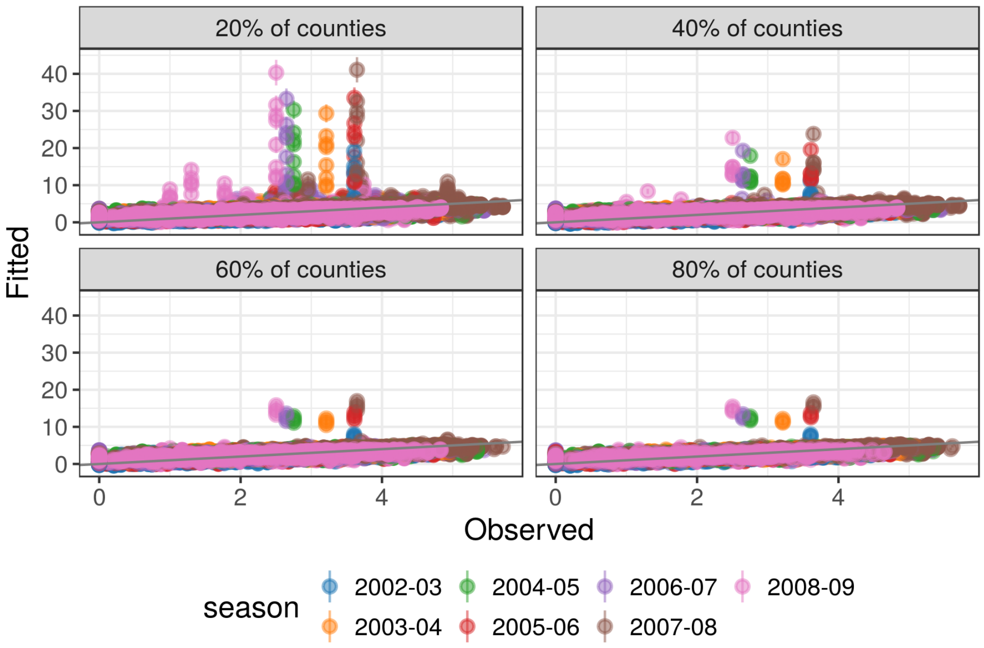

Figure W. Scatterplot between original observation and fitted value among observations that were excluded from fixed-location model replicates.

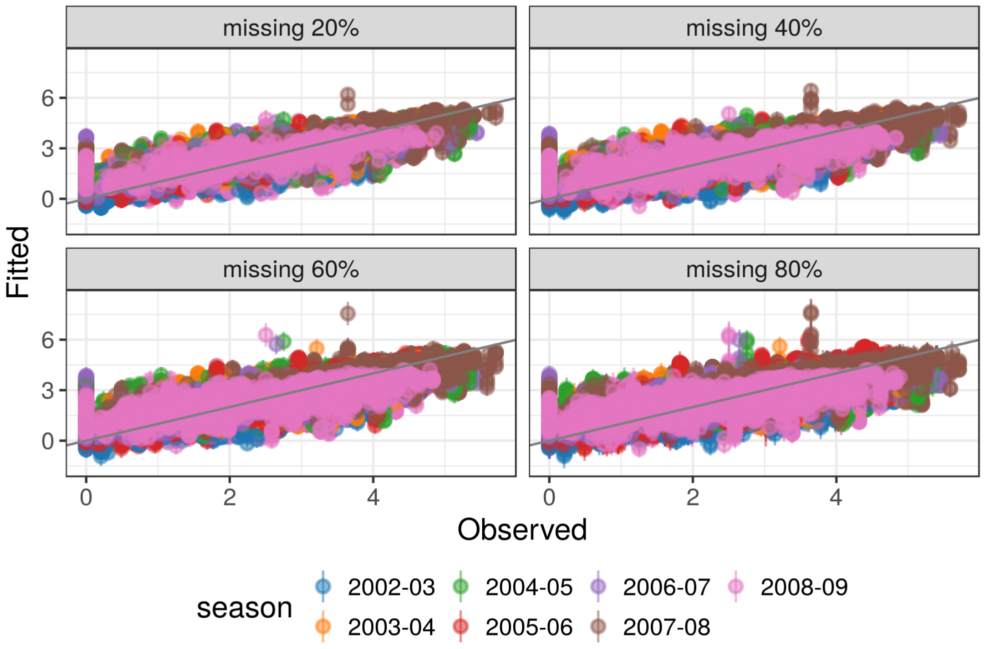

Figure X. Scatterplot between original observation and fitted value among observations that were excluded from moving-location model replicates.

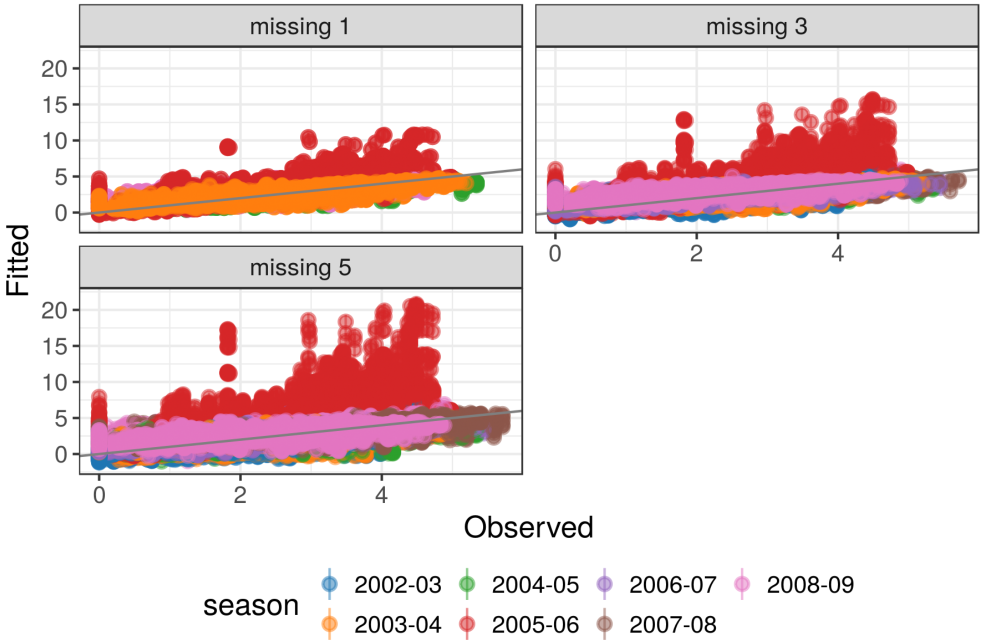

Figure Y. Scatterplot between original observation and fitted value among observations that were excluded from historical-season model replicates.

Group effect	Mean of the precision estimate
Season	18.00
Region	137.78
State	113.46
County	29,988.82
County spatial dependence	13.32
Observation error	14.00

Table D. Comparison of the mean precision estimates for group effects in total epidemic duration models, where precision is the inverse of the variance.

4 Epidemic duration surveillance models

4.1 Surveillance model and spatial predictors

We assessed model diagnostics for the total population model for epidemic duration (Figure Z, Figure AA). We examined the mean precision estimates for the group effects —season, region, state, county, county spatial dependence, observation error —to identify which effects explained most of the variability across observations (Table D).

Socio-environmental and measurement drivers

We incorporated socio-environmental and measurement-related determinants into the total population epidemic duration model (Figure AB, Figure AC).

4.2 Influenza surveillance and spatial patterns

We examined the spatial and temporal patterns for the epidemic duration model (Figure 2, Figure AD, Figure AE, Figure AF).

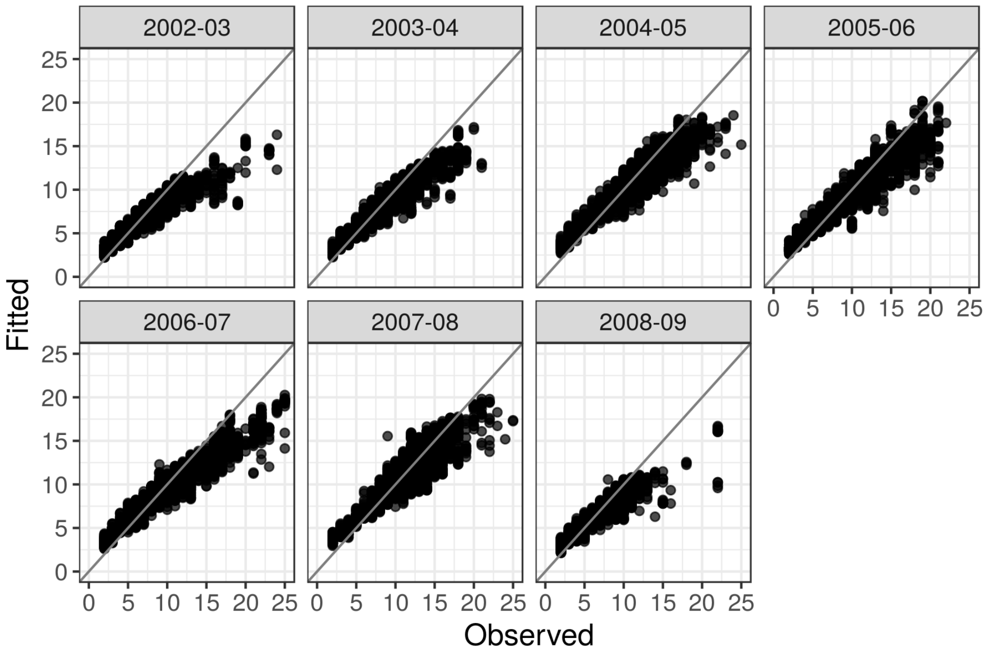

Figure Z. Observed versus fitted means for epidemic duration in weeks (Pearson's $R = 0.71$). The 45 degree line is in grey.

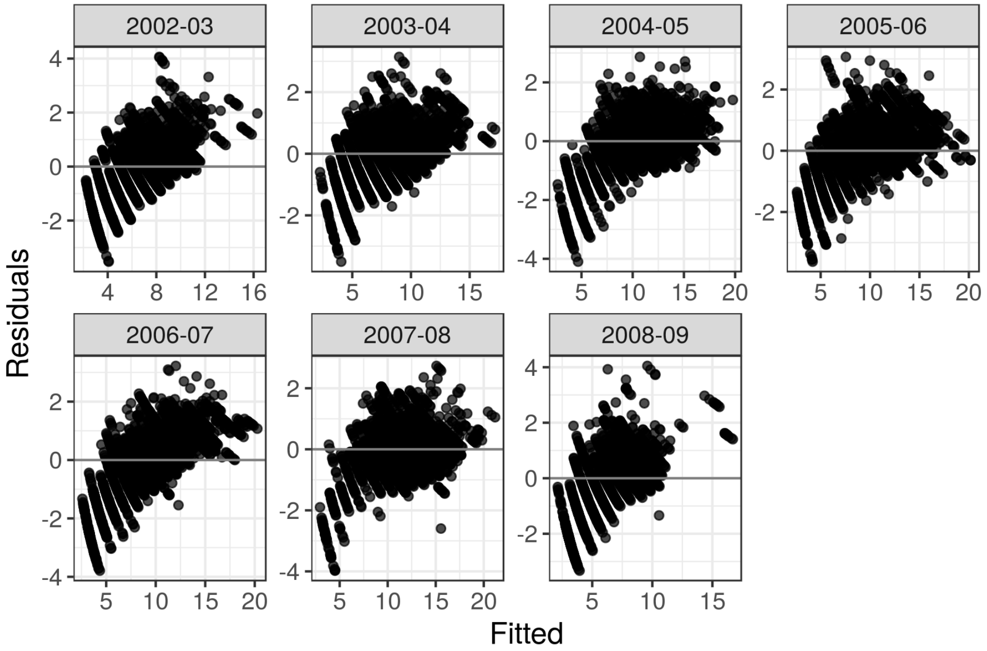

Figure AA. Residuals versus fitted values for epidemic duration.

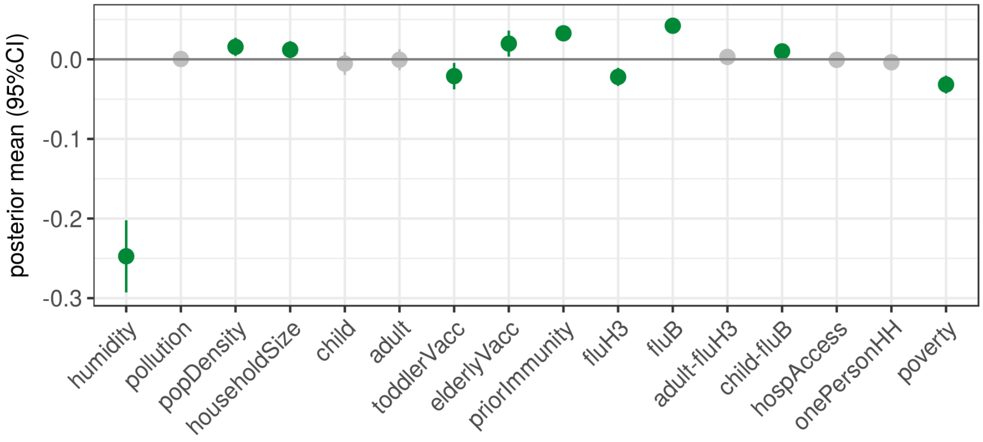

Figure AB. For the total population epidemic duration models, these are the 95% credible intervals for the posterior distributions of the socio-environmental coefficients.

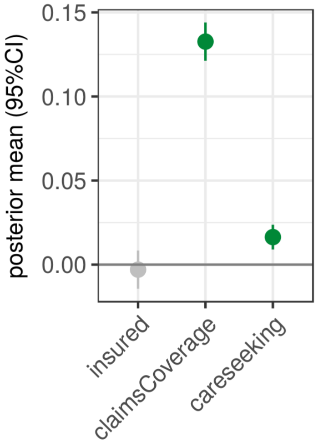

Figure AC. For the total population epidemic duration models, these are the 95% credible intervals for the posterior distributions of measurement-related coefficients.

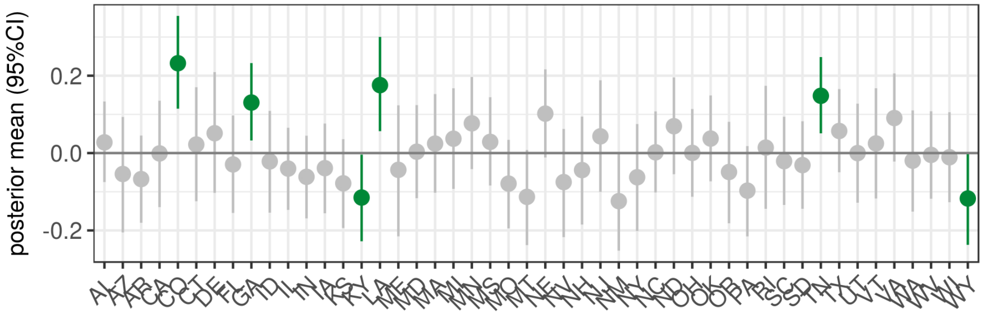

Figure AD. 95% credible intervals for state group effects for the epidemic duration model.

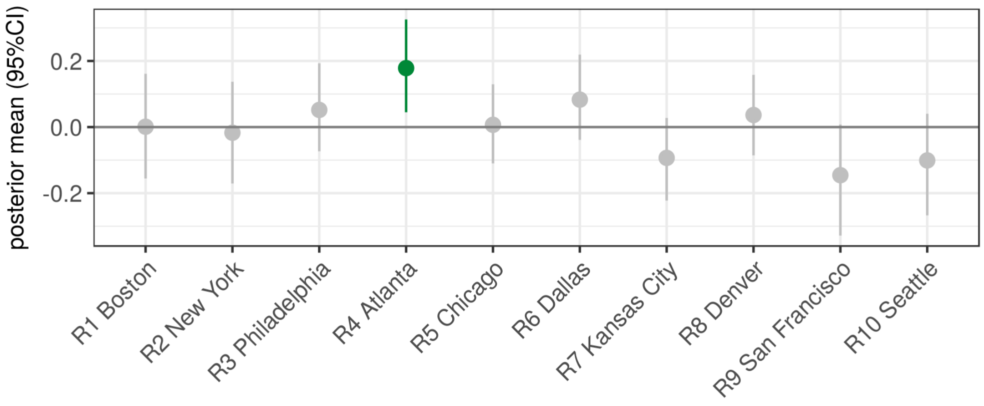

Figure AE. 95% credible intervals for HHS region group effects for the epidemic duration model.

4.3 Comparison of epidemic duration and epidemic intensity

We compared the epidemic duration and epidemic intensity fitted values and observations and fitted values (Figure AG).

5 Epidemic intensity surveillance models for children and adults

Children and adults comprise the largest components of the U.S. population, and many studies have considered shifts in immunity due to differences in contact patterns, shifting risk between children and adults over time, interactions between influenza types/subtypes by age, and differences in vaccine effectiveness by age group[4–9]. Considering the potential to elucidate age-specific processes and improve targeting of public health interventions, we used the multi-season model to examine drivers of epidemic intensity in the child and adult populations.

5.1 Surveillance model and spatial predictors

Both models appeared to fit the observed epidemic intensity well. The Pearson's cross-correlation coefficient between the observed and fitted mean relative risk was $R = 0.89$ and $R = 0.90$ for the child and adult epidemic intensity models, respectively (Figure AH). For both age groups,

Socio-environmental and measurement factors

In reference to the total epidemic intensity results, the child and adult models shared the same significant positive associations for the interaction term between child population and influenza B circulation and a proxy for prior immunity, and the same significant negative associations for adult and child population sizes, average flu season specific humidity, proportion of single person households, and infant vaccination coverage (Figure AI). The child and adult models shared a positive association with hospitals per capita where the total population model had no effect, and a negative association with estimated average household size where the total population model had a positive effect.

Child population epidemic intensity had a unique positive association with influenza B circulation and a unique negative association with elderly vaccination coverage. Adult population epidemic intensity had unique positive associations with H3 circulation among influenza A, proportion of the population in poverty, and elderly vaccination coverage, and a unique negative association with the interaction between adult and influenza H3.

Similar to the total population models, the child and adult epidemic intensity models had significant positive associations with care-seeking behavior and claims database coverage. However, both the child and adult epidemic intensity models had significant negative associations with proportion of the population with health insurance, where the total population model demonstrated no effect.

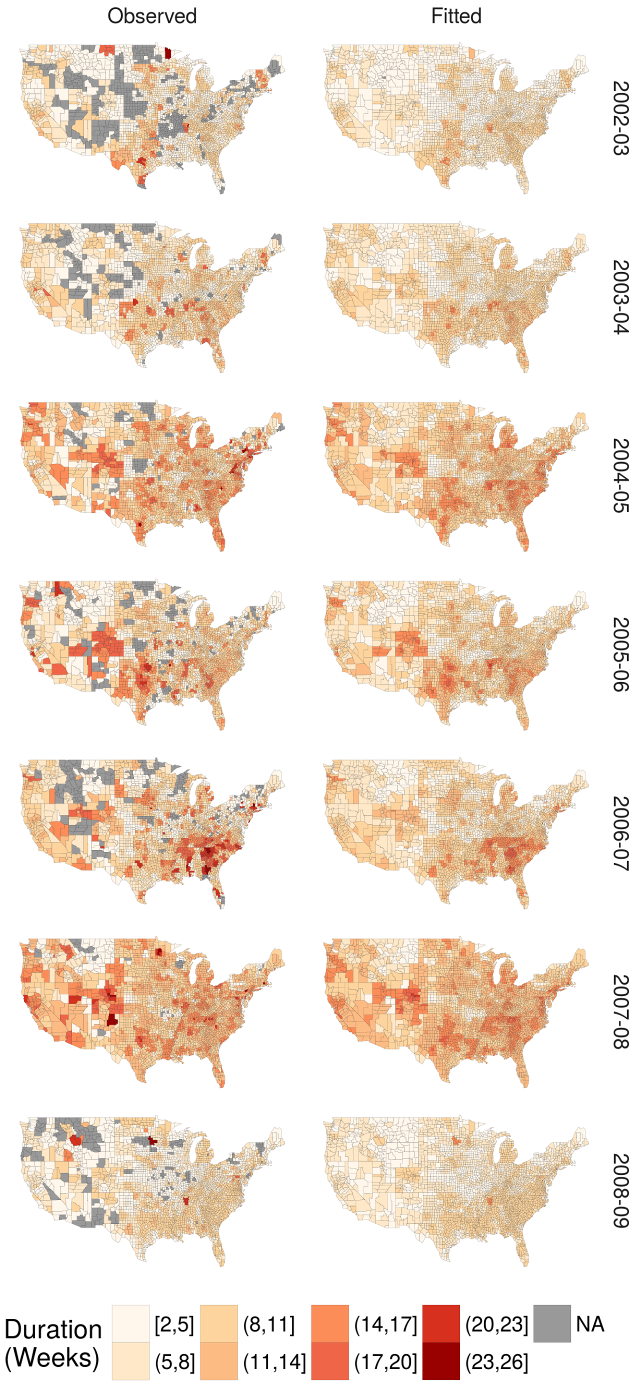

Figure AF. Continental U.S. county maps for observed (left) and fitted (right) epidemic duration in weeks from 2002-03 through 2008-09.

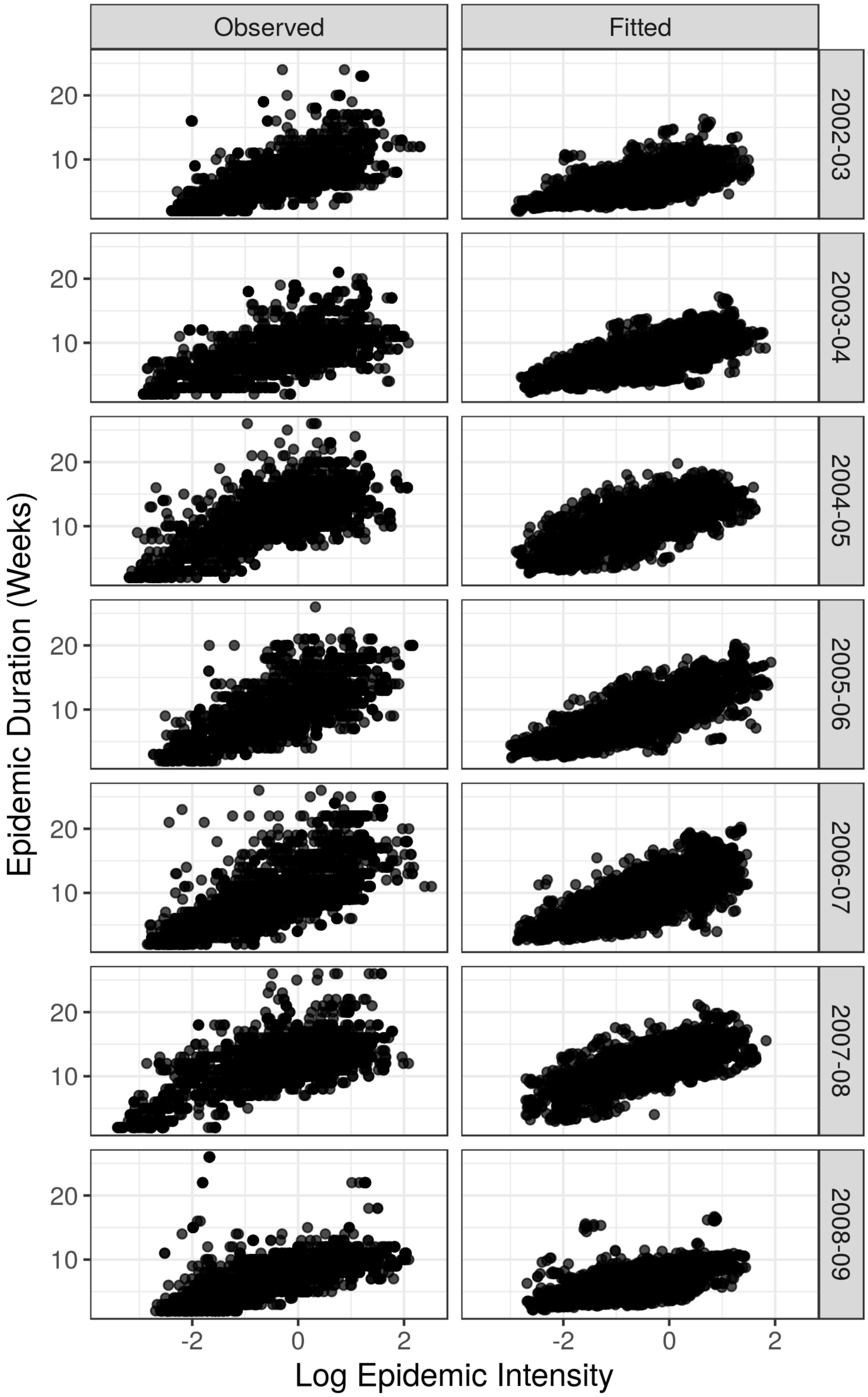

Figure AG. Comparison of epidemic duration and log epidemic intensity among observed (left) and fitted (right) values.

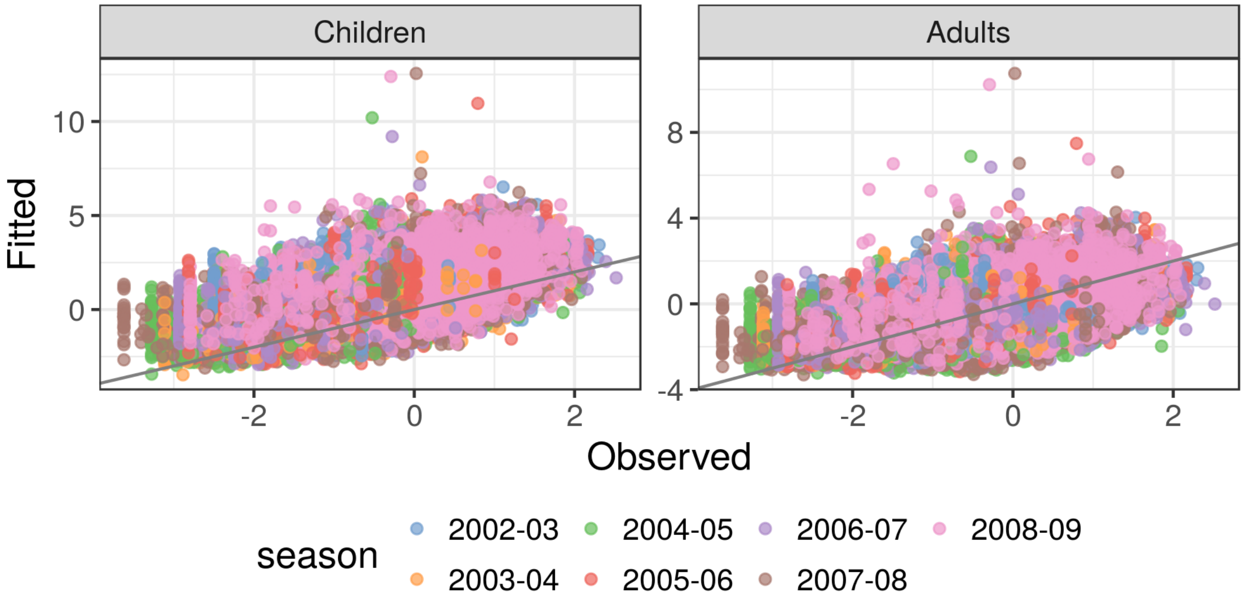

Figure AH. Comparison of observed and fitted mean relative risk of epidemic intensity across flu seasons from 2002-2003 through 2008-2009 for children (Pearson's $R = 0.89$) and adults (Pearson's $R = 0.90$).

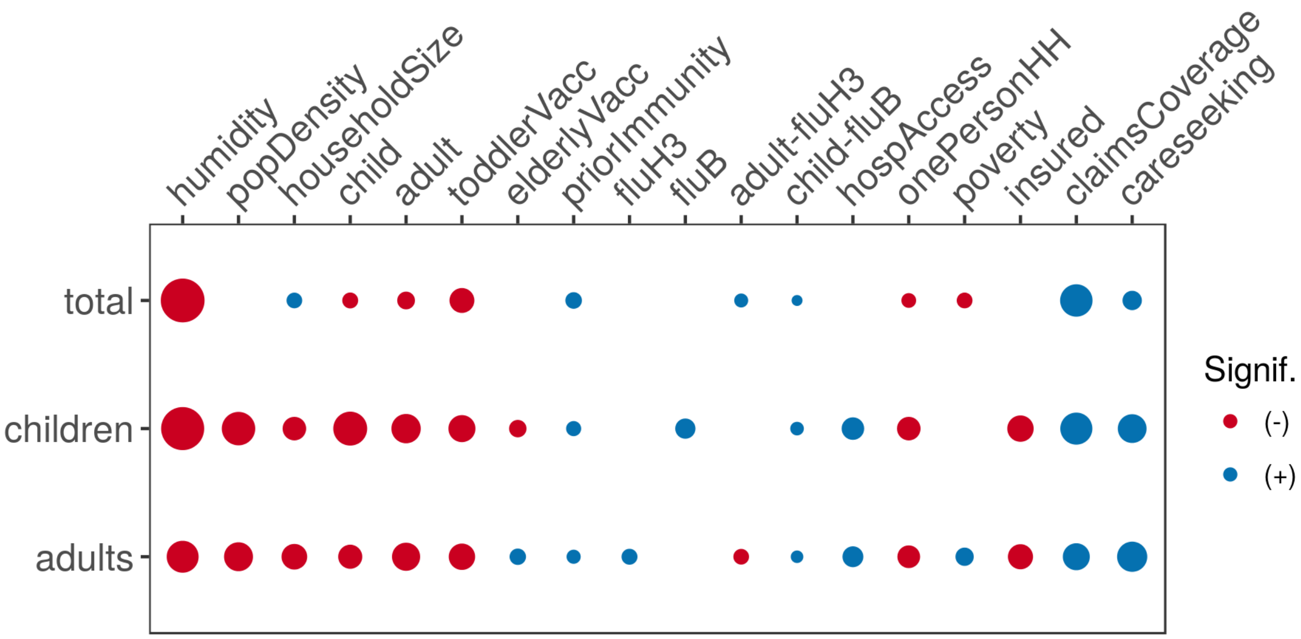

Figure AI. Diagram comparing model inference between total, child, and adult seasonal intensity. Predictors with no significant effect across the three models were removed for viewing ease, and absence of a dot means the effect was not significant.

Predictor	VIF
humidity	1.78
pollution	1.42
popDensity	1.16
householdSize	2.07
child	2.93
adult	2.07
toddlerVacc	1.66
elderlyVacc	1.84
priorImmunity	1.03
fluH3	1.59
fluB	1.19
adult-fluH3	1.10
child-fluB	1.01
hospAccess	1.33
onePersonHH	1.79
poverty	1.44
insured	1.78
claimsCoverage	1.51
careseeking	1.07

Table E. Variance Inflation Factors among final model predictors.

5.2 Influenza surveillance and spatial patterns

We examined the spatial patterns for the child and adult epidemic intensity models (Figure AJ, Figure AK). Similar to results for the total population, several South Atlantic states had greater risk while Plains states had lower risk of epidemic intensity for both children and adults (Figure AL, Figure AM).

6 Model predictors

6.1 Checks for multicollinearity

We checked for multicollinearity among predictors by examining Spearman rank cross-correlation coefficients between all pairs of final model predictors, excluding interaction terms (Figure AN). No single pair had a linear correlation coefficient that exceeded a magnitude of 0.6.

We also assessed multicollinearity among the final set of predictors in our model according to the rule of thumb that Variance Inflation Factors (VIFs) should be less than 10. No predictors violated this rule of thumb and VIFs are listed in table Table E.

Additionally, we ran our epidemic intensity model with each coefficient individually (Figure AO). Multicollinearity between predictors may sometimes be detected when a predictor significantly deviates from zero in the single predictor model, but does not appear to have an effect in a multivariate context. Some predictors (pollution, popDensity, fluB) that were significant in the single predictor context no longer had an effect in our complete model (and vice versa for householdSize and child). Nevertheless, all of these predictors had small effect sizes in both single variable and multivariate models, and the other predictors that were significant in both models retained effect sizes with the same order of magnitude and directionality.

6.2 Medical claims coverage

Medical claims database coverage increased over time across each state (Figure AP).

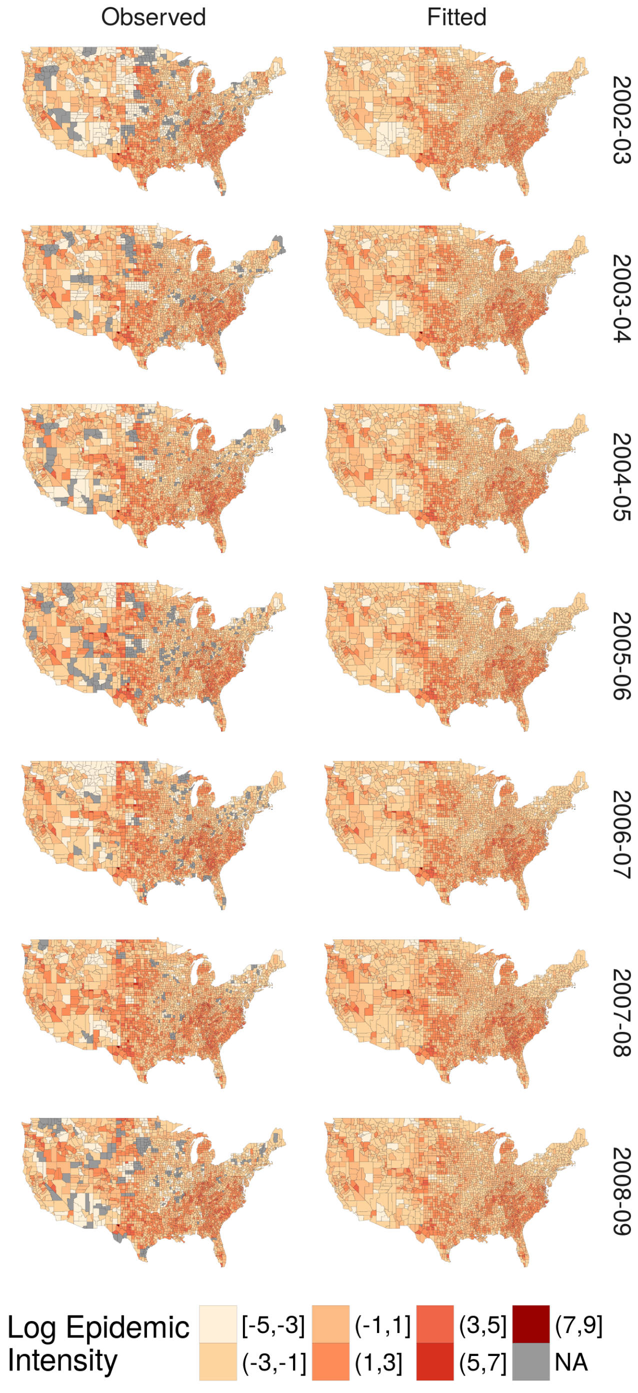

Figure AJ. Continental U.S. county maps for observed (left) and fitted (right) log epidemic intensity among children in weeks from 2002-03 through 2008-09.

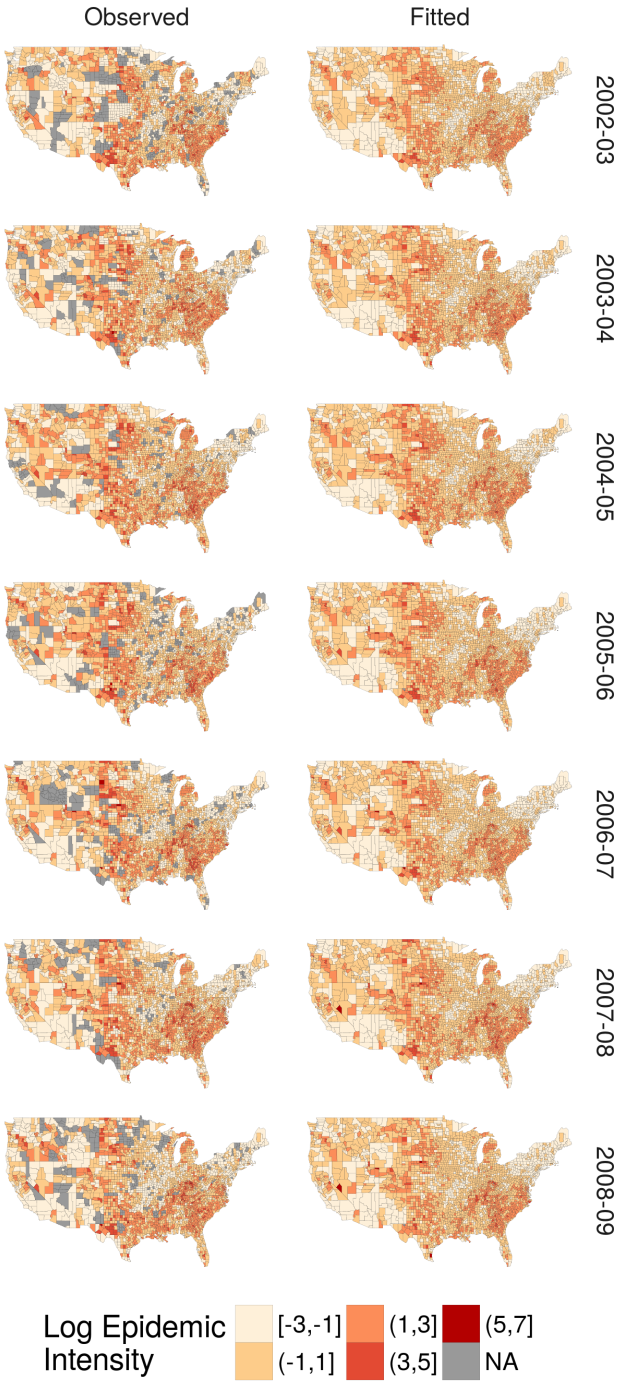

Figure AK. Continental U.S. county maps for observed (left) and fitted (right) log epidemic intensity among adults in weeks from 2002-03 through 2008-09.

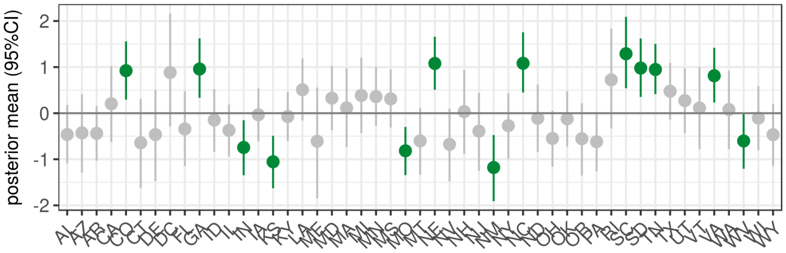

Figure AL. 95% credible intervals for state group effects for the child epidemic intensity model.

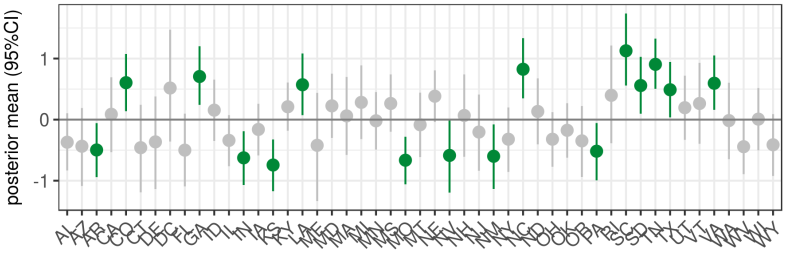

Figure AM. 95% credible intervals for state group effects for the adult epidemic intensity model.

7 Data processing

7.1 Defining influenza disease burden

Disease burden was assessed after processing the medical claims data as described in the Methods section. Time series data detrending and identification of the influenza epidemic period are graphically depicted in Figure AQ.

7.2 Prior immunity tables

For seasonal influenza years, we calculated a proxy for prior immunity that is described in detail in the Methods. Here, we present our proxy for the proportion of individuals infected in the previous influenza season that would have protection during the current season, after accounting for the distribution of circulating influenza strains (Table F). As strain circulation is reported by CDC by HHS regions, that is the spatial resolution of these data.

HHS Region	1	2	3	4	5	6	7	8	9	10
2002-2003	.19	.15	.095	.14	.29	.066	.13	.30	.26	.22
2003-2004	8E-4	0	7E-4	0	0	0	0	0	0	0
2004-2005	4E-6	0	7E-6	0	0	0	0	0	0	0
2005-2006	.63	.63	.43	.67	.58	.55	.66	.50	.35	.46
2006-2007	.037	.059	.13	.043	.053	.065	.084	.020	.061	.021
2007-2008	0	0	0	0	0	0	0	0	0	0
2008-2009	.028	.029	.015	.055	.011	.022	.018	.012	.048	.018

Table F. Population-weighted average for a proxy of prior immunity that captures the proportion of individuals infected in the previous flu season that would have protection during the current flu season, accounting for the distribution of circulating flu strains. The prior immunity model predictor was calculated as the product of the below proportions and the seasonal intensity of the immediately preceding influenza season in a given county.

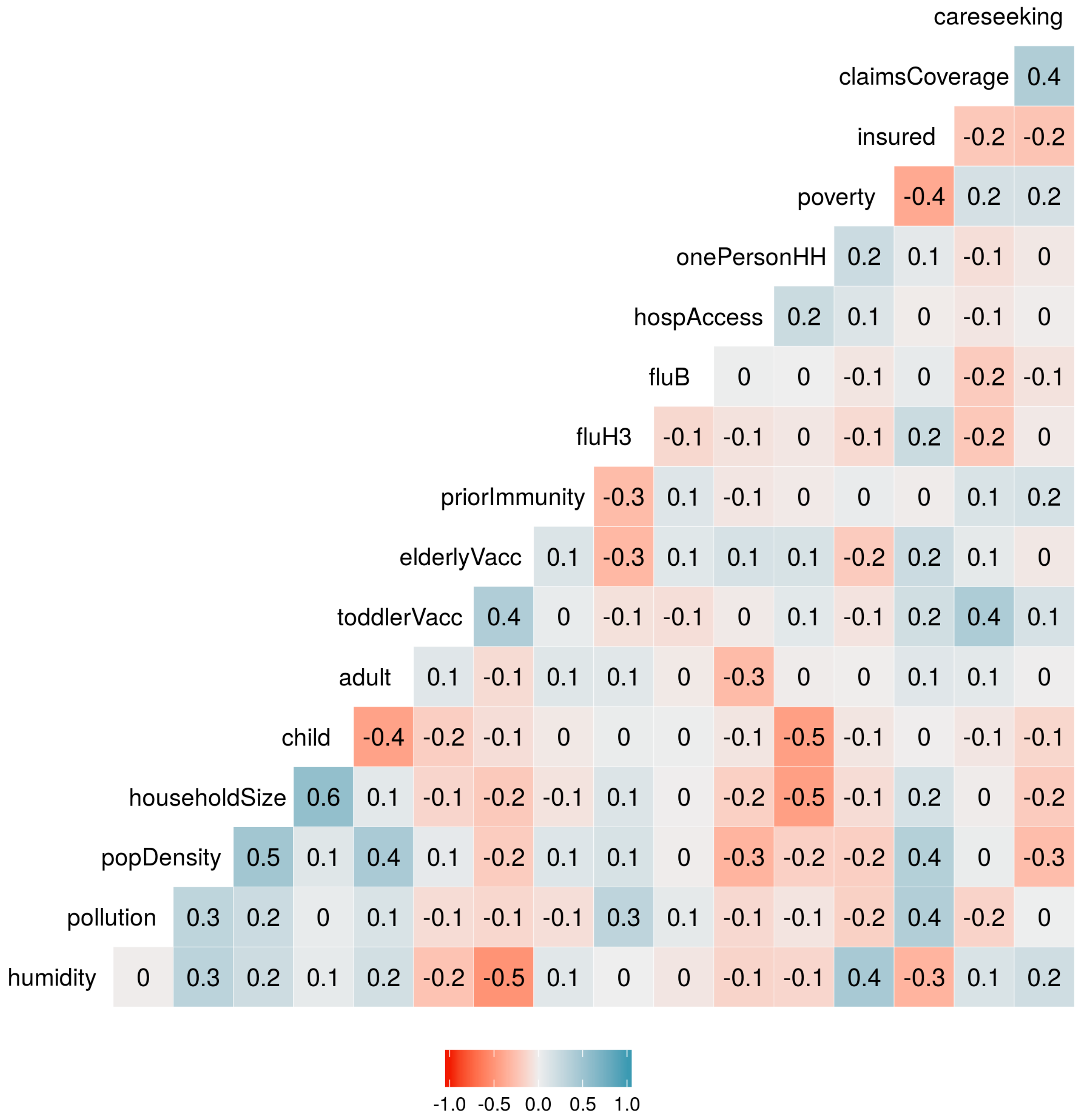

Figure AN. Spearman rank cross-correlation matrix for all pairs of final model predictors.

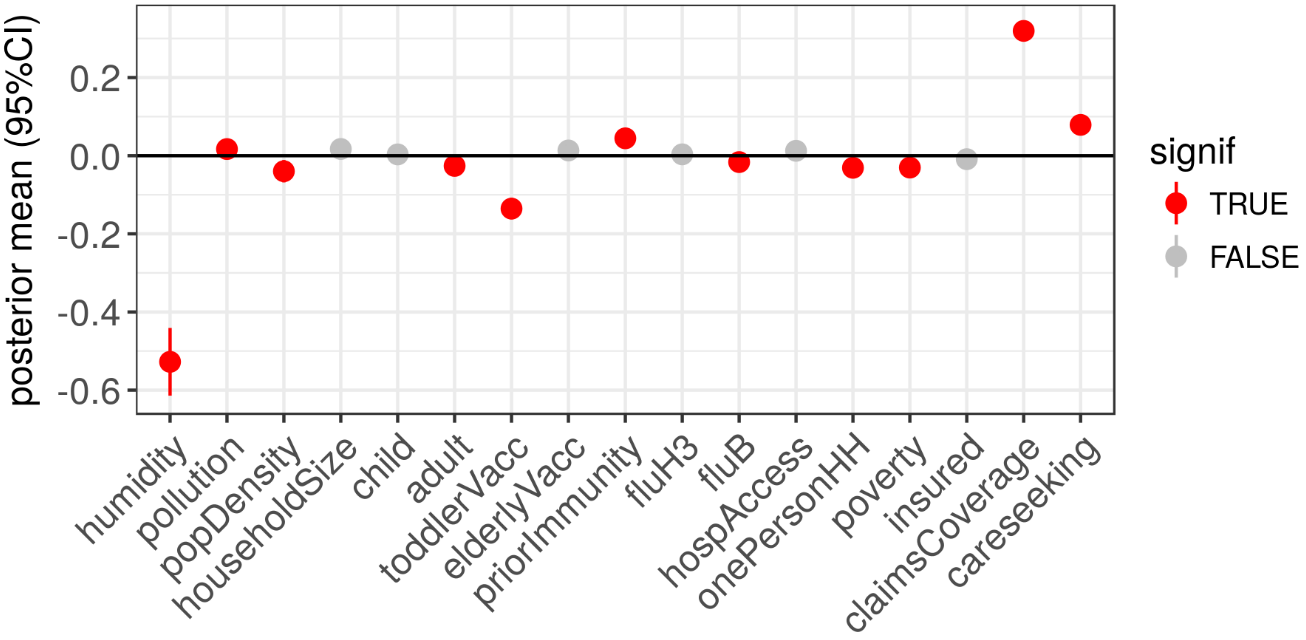

Figure AO. These are the 95% credible intervals among multi-season models with a single predictor for epidemic intensity.

7.3 Comparison to alternative data processing steps

Due to concerns regarding spatial variation in care-seeking, we processed the medical claims data with an alternative ILI measure (ILI ratio), which was defined as the percentage of total visits due to ILI (ILI/total visits * 100). This alternative measures, due to its normalization by total visits, adjusts for observation biases due to insurance coverage, medical claims database coverage, care-seeking behavior, and population size. We investigated the Spearman rank correlation coefficients between our primary ILI measure, which is population-normalized, and ILI ratio, and found a strong positive correlation between the two (ρ = 0.79, p-value ¡ 2.2E-16) .

References

1. Viboud, C. *et al.* Demonstrating the use of high-volume electronic medical claims data to monitor local and regional influenza activity in the US. *PLoS One* **9**, e102429 (2014). DOI 10.1371/journal.pone.0102429.
2. Centers for Disease Control and Prevention. Interim results: state-specific influenza A (H1N1) 2009 monovalent vaccination coverage - United States, October 2009-January 2010. *Morb. Mortal. Wkly. Rep.* **59**, 363–8 (2010). DOI mm5912a2 [pii].
3. Centers for Disease Control and Prevention. Serum Cross-Reactive Antibody Response to a Novel Influenza A (H1N1) Virus After Vaccination with Seasonal Influenza Vaccine. *Morb. Mortal. Wkly. Rep.* **58**, 521–524 (2009).
4. Bansal, S., Pourbohloul, B., Hupert, N., Grenfell, B. & Meyers, L. A. The shifting demographic landscape of pandemic influenza. *PLoS One* **5**, e9360 (2010). DOI 10.1371/journal.pone.0009360.
5. Lee, E. C., Viboud, C., Simonsen, L., Khan, F. & Bansal, S. Detecting Signals of Seasonal Influenza Severity through Age Dynamics. *BMC Infect. Dis.* **15** (2015). DOI 10.1186/s12879-015-1318-9.
6. Ewing, A., Lee, E. C., Viboud, C. & Bansal, S. Contact, travel, and transmission: The impact of winter holidays on influenza dynamics in the United States. *J. Infect. Dis.* (2016). DOI https://doi.org/10.1093/infdis/jiw642.
7. Schanzer, D., Vachon, J. & Pelletier, L. Age-specific differences in influenza A epidemic curves: do children drive the spread of influenza epidemics? *Am. J. Epidemiol.* **174**, 109–17 (2011). DOI 10.1093/aje/kwr037.

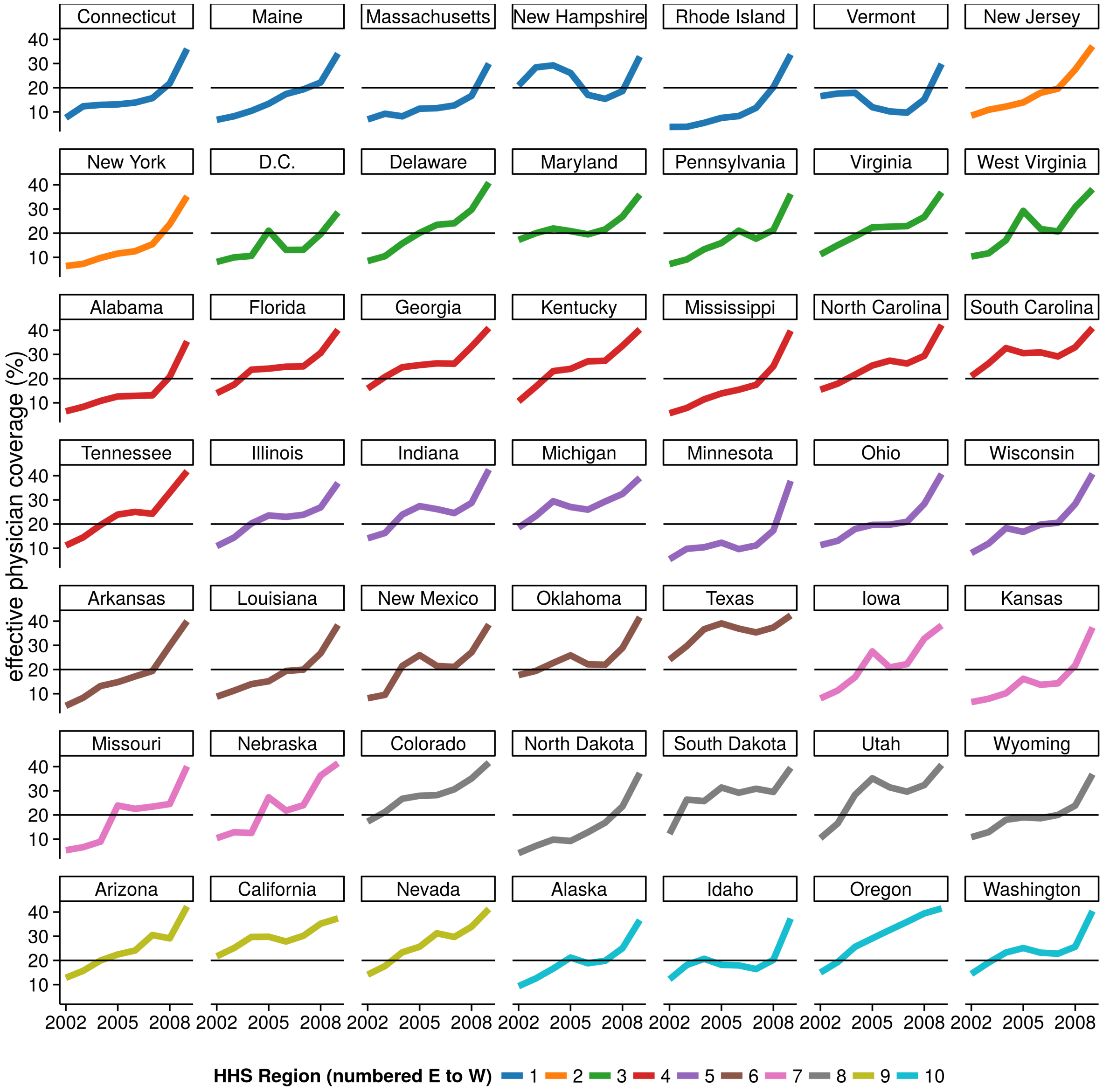

Figure AP. Medical claims database coverage by year and state. Colors represent states that belong to the same HHS region. The black horizontal line at 20% effective physician coverage is a visual guide to ease the comparison of data across panels.

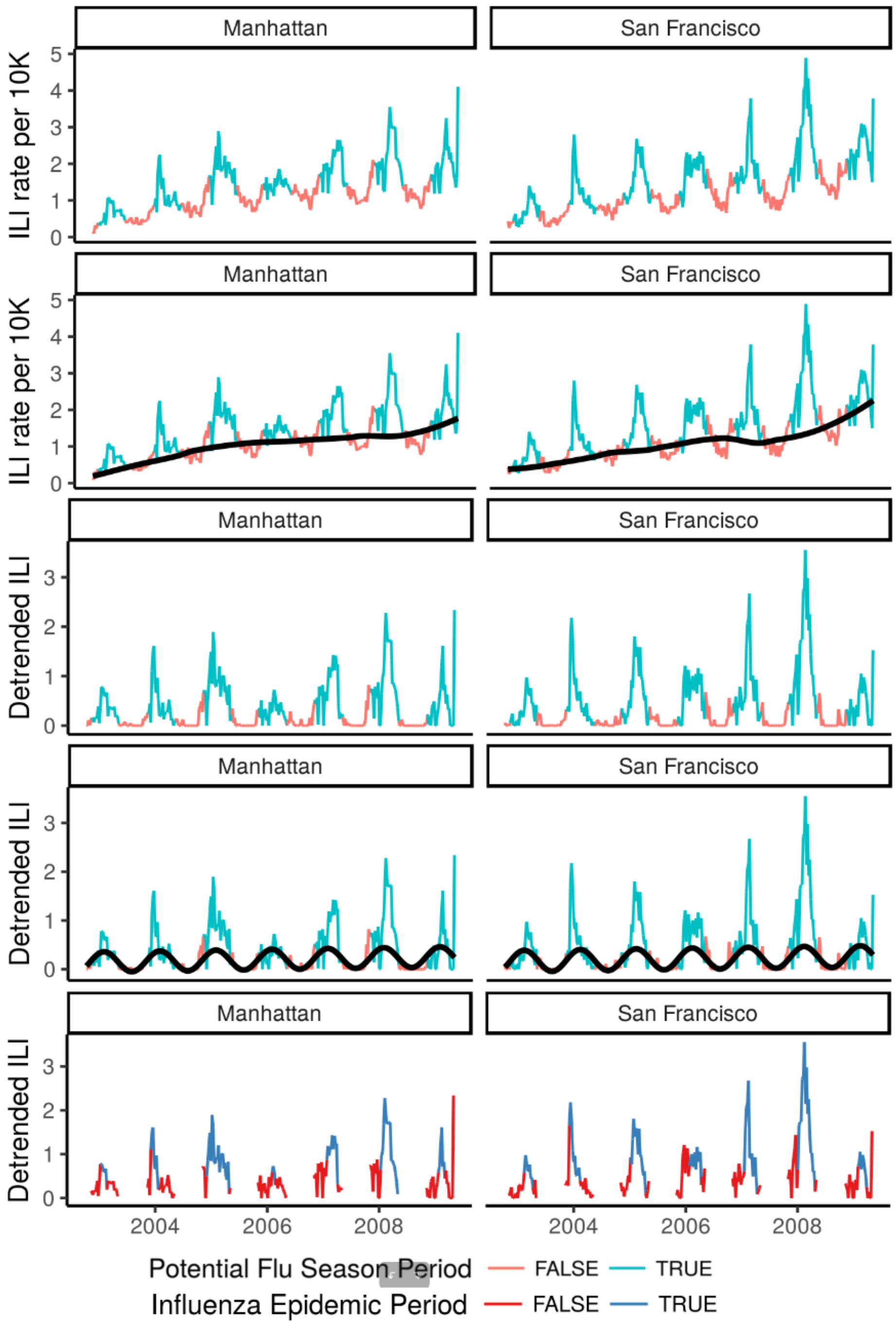

Figure AQ. Graphical representation of the data processing steps performed for two example county-level time series (top to bottom). ILI rate per 10K represents the raw ILI reports in the medical claims (first row of figures). These data were detrended through subtraction according to the LOESS fit (black line in second row) to get the detrended ILI (third row). All negative values were replaced with zero among the detrended ILI. A seasonal regression model (black line in fourth row) was fit to weeks outside of the potential flu season period (salmon segments in fourth row) and used to identify the true influenza epidemic period for each county (blue segments in fifth row).

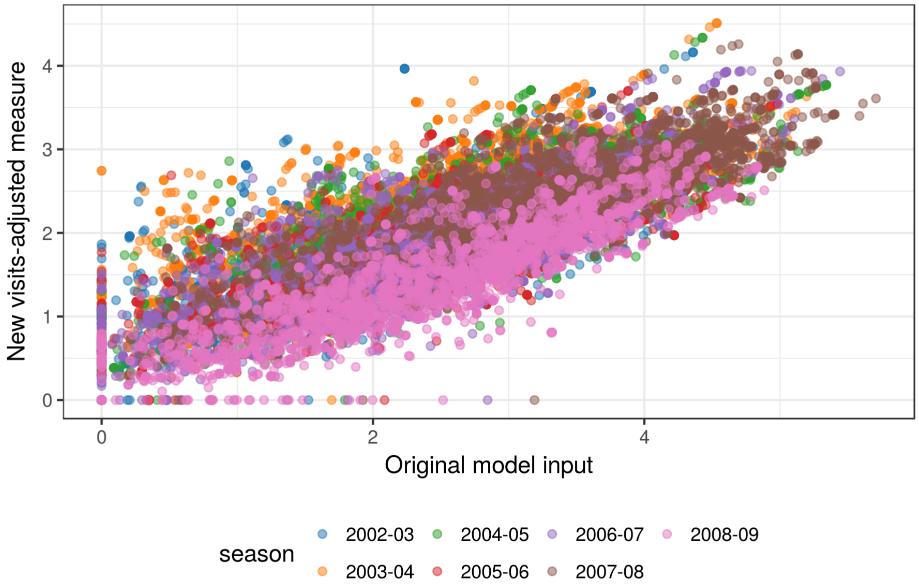

Figure AR. Graphical representation of the data processing steps performed for two example county-level time series (top to bottom). ILI rate per 10K represents the raw ILI reports in the medical claims (first row of figures). These data were detrended through subtraction according to the LOESS fit (black line in second row) to get the detrended ILI (third row). All negative values were replaced with zero among the detrended ILI. A seasonal regression model (black line in fourth row) was fit to weeks outside of the potential flu season period (salmon segments in fourth row) and used to identify the true influenza epidemic period for each county (blue segments in fifth row).

8. Gostic, K. M., Ambrose, M., Worobey, M. & Lloyd-Smith, J. O. Potent protection against H5N1 and H7N9 influenza via childhood hemagglutinin imprinting. *Sci. (80-.).* **354**, 722–726 (2016). DOI 10.1126/science.aag1322.

9. Khiabanian, H., Farrell, G. M., St George, K. & Rabadan, R. Differences in patient age distribution between influenza A subtypes. *PLoS One* **4**, e6832 (2009). DOI 10.1371/journal.pone.0006832.
